# In silico study of subtilisin-like protease 1 (SUB1) from different *Plasmodium* species in complex with peptidyl-difluorostatones and characterization of potent pan-SUB1 inhibitors

**DOI:** 10.1016/j.jmgm.2016.01.005

**Published:** 2016-03

**Authors:** Simone Brogi, Simone Giovani, Margherita Brindisi, Sandra Gemma, Ettore Novellino, Giuseppe Campiani, Michael J. Blackman, Stefania Butini

**Affiliations:** a European Research Centre for Drug Discovery and Development (NatSynDrugs), University of Siena, via Aldo Moro 2, 53100, Siena, Italy; b Dipartimento di Biotecnologie, Chimica e Farmacia, University of Siena, via Aldo Moro 2, 53100, Siena, Italy; c Centro Interuniversitario di Ricerche sulla Malaria (CIRM), University of Perugia, Perugia, Italy; d Dipartimento di Farmacia, University of Naples Federico II, Via D. Montesano 49, 80131, Naples, Italy; e Division of Parasitology, MRC National Institute for Medical Research, Mill Hill, London NW7 1AA, UK

**Keywords:** Homology modeling, Molecular docking, Subtilisin-like protease, Malaria, Difluorostatone-based inhibitors, Pharmacophore modeling

## Abstract

*Plasmodium falciparum* subtilisin-like protease 1 (SUB1) is a novel target for the development of innovative antimalarials. We recently described the first potent difluorostatone-based inhibitors of the enzyme ((4*S*)-(*N*-((*N*-acetyl-l-lysyl)-l-isoleucyl-l-threonyl-l-alanyl)-2,2-difluoro-3-oxo-4-aminopentanoyl)glycine (**1**) and (4*S*)-(*N*-((*N*-acetyl-l-isoleucyl)-l-threonyl-l-alanylamino)-2,2-difluoro-3-oxo-4-aminopentanoyl)glycine (**2**)). As a continuation of our efforts towards the definition of the molecular determinants of enzyme-inhibitor interaction, we herein propose the first comprehensive computational investigation of the SUB1 catalytic core from six different *Plasmodium* species, using homology modeling and molecular docking approaches. Investigation of the differences in the binding sites as well as the interactions of our inhibitors **1**,**2** with all SUB1 orthologues, allowed us to highlight the structurally relevant regions of the enzyme that could be targeted for developing pan-SUB1 inhibitors. According to our in silico predictions, compounds **1,2** have been demonstrated to be potent inhibitors of SUB1 from all three major clinically relevant *Plasmodium* species (*P. falciparum*, *P. vivax*, and *P. knowlesi*). We next derived multiple structure-based pharmacophore models that were combined in an inclusive pan-SUB1 pharmacophore (SUB1-PHA). This latter was validated by applying in silico methods, showing that it may be useful for the future development of potent antimalarial agents.

## Highlights

• Homology models of four SUB1 orthologues from *P. falciparum* species were produced.
• We analyzed the binding mode of our previous difluorostatone inhibitors to six SUB1.
• In vitro activity of our difluorostatone-based inhibitors was correctly predicted.
• We derived a structure-based pan-SUB1 pharmacophore, and validated it in silico.
• We confirmed that development of pan-SUB1 inhibitors is a feasible task.

## Graphical abstract

**Figure:**
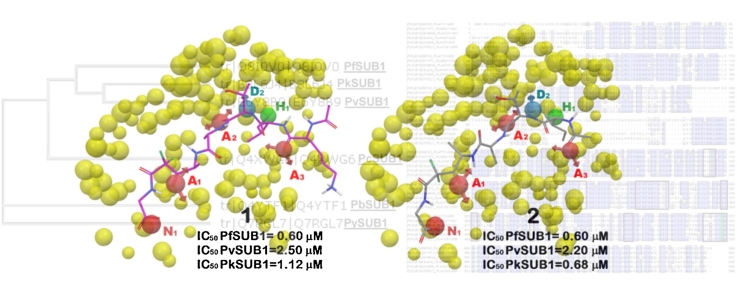

## 1 Introduction

**Fig. 1:**
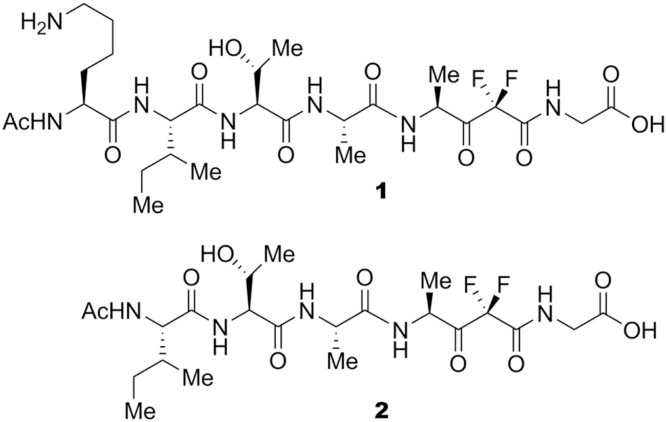
Difluorostatone-based inhibitors **1** (4*S*)-(*N*-((*N*-acetyl-l-lysyl)-l-isoleucyl-l-threonyl-l-alanyl)-2,2-difluoro-3-oxo-4-aminopentanoyl)glycine) and **2** ((4*S*)-(*N*-((*N*-acetyl-l-isoleucyl)-l-threonyl-l-alanyl amino)-2,2-difluoro-3-oxo-4-aminopentanoyl)glycine).

Several *Plasmodium* species cause malaria in humans. Among them, *P. falciparum* is the etiological agent of the most deadly form of malaria. As a consequence, much attention has been devoted to the search for novel drugs for treating *P. falciparum* infections. *P. vivax* has historically been considered relatively avirulent compared to *P. falciparum*, so development of new chemotherapies against *P. vivax* has been relatively neglected [1]. However, morbidity due to *P. vivax* infection contributes to most of the social and economic burden of malaria outside Africa, and infections are complicated by relapses that can occur as much as 2 years following primary infection. In addition, it is now recognized that human infections by the zoonotic pathogen *P. knowlesi* are widespread in areas of South-East Asia [2]. Malaria caused by *P. knowlesi* can be severe and often fatal, so development of diagnostic tools and specific chemotherapies is urgently required. The *P. falciparum* subtilisin-like protease 1 (PfSUB1) is a serine protease which plays a key role in both egress of merozoites from infected erythrocytes and priming the developing merozoites for invasion of new erythrocytes [3], [4], [5], [6]. This enzyme also plays an essential role in the development and egress of hepatic merozoites [7], [8]. Drugs based on inhibitors of SUB1 could overcome the issue of resistance to chloroquine and several other currently available antimalarials, as well as the emerging resistance of *P. falciparum* to artemisinins [9], [10]. Moreover, the same approach can be exploited for the development of new chemotherapeutics against *P. vivax* and *P. knowlesi*, which express orthologous SUB1 enzymes [11]. It is worth noticing that, differently from other drug targets in malaria in which rapid selection of mutants was observed (e.g., cytochrome b targeted by atovaquone [12], [13], [14], [15]), PfSUB1 represents a particularly excellent drug target because the likelihood of simultaneous compensatory mutations in both the protease active site and the substrate cleavage sites that might result in drug resistance is low. Endogenous substrates of PfSUB1 have been investigated and some studies analyzing in silico the interaction of peptides based on endogenous sequences with PfSUB1 and PvSUB1 have been previously in depth analyzed [6], [11], [16], [17], [18]. Few PfSUB1 or PvSUB1 inhibitors have been described to date [11], [16], [19], [20]. We recently developed the first potent difluorostatone-based inhibitors (**1** and **2**, Fig. 1) of PfSUB1 [21] and we later in depth analyzed the structure-activity relationships (SARs) for this series of compounds [22]. In this context, our plan is the development of pan-inhibitors that may represent an innovative approach for treating infections caused by the human malaria pathogens.

Towards this ambitious aim, we decided to dissect the similarities between the SUB1 orthologues from all three major human malaria pathogens in order to ascertain the likelihood of developing a single inhibitor for all three enzymes. We here describe the development of a homology model of the active core of PkSUB1, and the comparison of the structural features of its binding site cleft with the crystal structures of PvSUB1 [23] and PfSUB1 [24]. To expand the scope of our investigation, we also developed homology models of SUB1 from *P. berghei*, *P. chabaudi*, and *P. yoelii*, three *Plasmodium* species that specifically infect rodents and are routinely used for testing antimalarial compounds in vivo. It has been previously demonstrated that the PbSUB1 active site is significantly different from that of PfSUB1 [11], so we extended our investigation to PcSUB1 and PySUB1. Moreover we have updated the PbSUB1 model previously described [11] using the experimentally solved PfSUB1 and PvSUB1 crystal structures as templates. The overall objective of the work here described is the analysis of the binding mode of our difluorostatone-based inhibitors to the six orthologous enzymes in order to: (i) assess the feasibility of a pan-inhibitor active against all three clinically relevant parasites; (ii) derive and validate a pharmacophore model to be used as design tool for the synthesis of pan-inhibitors and/or in a virtual screening campaign to identify novel chemical entities able to inhibit SUB1s, and (iii) verify the possibility of using the rodent malarial parasites as models to assess the efficacy of inhibitors designed on the basis of the human clinically relevant parasites.

## 2 Materials and methods

### 2.1 Difluorostatone-based inhibitors

Compounds **1** and **2** were synthesized following a previously described synthetic procedure [21] and were tested against Pv- and Pk-SUB1 as described in Paragraph 2.7.

### 2.2 Computational details

All the calculations performed in this work were carried out on three Cooler Master Centurion 5 (Intel Core2 Quad CPU Q6600 @ 2.40 GHz; Intel Core i5–2400CPU @ 3.10 GHz Quad; Intel Core i5–2500CPU @ 3.30 GHz Quad) with Ubuntu 10.04 LTS (long-term support) operating system running Maestro 9.2 (Schrödinger, LLC, New York, NY, 2011) and GOLD software (version 5.2, Cambridge Crystallographic Data Center, UK, 2013).

### 2.3 Homology modeling of SUB1

The sequence of SUB1s were taken in fasta format from UniProtKB [25] (PbSUB1 UniprotKB code: Q4YVE1; PySUB1 UniprotKB code: Q7RGL7; PcSUb1 UniprotKB code: Q4XWG6; PkSUB1 UniprotKB code: B3L6J4). The SUB1 homology models were built using the recently published PfSUB1 and PvSUB1 crystal structures (PDB codes: 4LVN and 4TR2, respectively) [23], [24], applying multiple template-based alignment as previously reported by us [21], [26], [27]. The sequence identity found by Prime during the template selection step for PbSUB1 were 4LVN 64%, 4TR2 58%; for PySUB1 were 4LVN 64%, 4TR2 58%; for PcSUB1 were 4LVN 67%, 4TR2 57%; and for PkSUB1 were 4LVN 75%, 4TR2 80%. In order to model the core catalytic domain of SUB1 orthologues Prime software [28] was used. Homology models were generated using the above-mentioned templates. These templates aligned to each query sequence were used for “Comparative Modeling” methods implemented in Prime. Since Prime offers several ways to build a model, we specified in the “build structure step” the method used for aligning multiple templates of all the SUB1 structures. Consensus model option was employed to build the model; this option allowed us to take into account all the previously selected templates since the model was built as an average of all templates. Each predicted SUB1 model for each different *Plasmodium* species was refined by means of Prime software by side-chain optimization and loop refinement. Further structure optimization was carried out using the MacroModel (MacroModel, version 9.9, Schrödinger, LLC, New York, NY, 2011) application implemented in Maestro suite 2011 using the Optimized Potentials for Liquid Simulations-all atom (OPLS-AA) force field 2005 with 10,000 maximum iterations and 0.001 as convergence threshold using PRCG method [29], [30].

The quality of modelled proteins was assessed by means of Ramachandran plots generated by the RAMPAGE webserver (http://mordred.bioc.cam.ac.uk/∼rapper/rampage.php accessed date May 2015) [31]. For all the modelled SUB1, around 95% of the protein residues lie in the favoured region of the plot, around 4% lie in the additional allowed region and less than 0.6% (amino acids not involved in the binding site) of the residues were located in the disallowed regions. As previously found for the PfSUB1 homology model [21] the other generated SUB1 3D structures displayed a satisfactory and similar stereochemical quality. Accordingly, the results of the RAMPAGE webserver revealed that over 99% of the residues of our refined SUB1 models sit in the allowed regions of the Ramachandran Plot. This value is higher than the cut-off value (96.1%) defined for the most reliable models [32]. Consequently, the stereochemical quality of our SUB1 homology models was acceptable, displaying a very low percentage of residues having phi/psi angles in outlier regions.

### 2.4 Molecular docking protocol

#### 2.4.1 Ligand preparation

Three-dimensional (3D) structure building for all compounds in this study was carried out using Maestro 9.2 (Schrödinger, LLC, New York, NY, 2011). The stereochemistry of compounds was taken into account according to Fig. 1. Molecular energy minimizations were performed in MacroModel using the Optimized Potentials for Liquid Simulations-all atom (OPLS-AA) force field 2005. Solvent effects were simulated using the analytical Generalized-Born/Surface-Area (GB/SA) model [33], and no cutoff for nonbonded interactions was selected. Polak-Ribiere conjugate gradient (PRCG) method with 1000 maximum iterations and 0.001 gradient convergence threshold was employed [34]. All the compounds reported in this paper were treated by the LigPrep application (version 2.5, Schrödinger, LLC, New York, NY, 2011), implemented in Maestro suite 2011, generating the most probable ionization state of any possible enantiomers and tautomers at cellular pH values (7 ± 0.5).

#### 2.4.2 Protein preparation

The SUB1 homology models and crystal structures were imported into Schrödinger Maestro molecular modeling environment (Maestro, version 9.2; Schrödinger, LLC: New York, 2011). For the crystal structures all the water molecules and the chemicals used for the crystallization procedure were removed. Resulting crystal structures and homology models were submitted to the protein preparation wizard workflow implemented in Maestro suite 2011 (Protein Preparation Wizard workflow 2011; http://www.schrodinger.com/supportdocs/18/16). This protocol allowed us to obtain a reasonable starting structure of proteins for molecular docking calculations by a series of computational steps. In particular, we performed three steps to [1] add hydrogens, [2] optimize the orientation of hydroxyl groups, Asn, and Gln, and the protonation state of His, and [3] perform a constrained refinement with the impref utility, setting the max RMSD of 0.30. The impref utility consists of a cycle of energy minimization based on the impact molecular mechanics engine and on the OPLS_2005 force field [29], [30].

#### 2.4.3 Molecular docking

Molecular Docking studies were carried out using GOLD 5.2 (Genetic Optimization for Ligand Docking) software from the Cambridge Crystallographic Data Center, UK, that uses the Genetic algorithm (GA) [35]. This method allows a partial flexibility of protein and full flexibility of ligand. For each of the 100 independent GA runs, a maximum number of 125000 GA operations were performed. The search efficiency values were set at 200% in order to increase the flexibility of the ligands docked. As reported in the GOLD user manual this parameter is recommended for large, highly flexible ligands. The active site radius of 8 Å was chosen by XYZ coordinates from the center of the catalytic triad, considering the catalytic Ser as previously described [21]. Default cutoff values of 2.5 Å (dH-X) for hydrogen bonds and 4.0 Å for van der Waals distance were employed. When the top three solutions attained RMSD values within 1.5 Å, GA docking was terminated. The fitness function GoldScore [36] was evaluated. All the poses herein presented are representative of the most populated clusters of docked solutions, and were chosen after cluster analysis and visual inspection.

### 2.5 Structure-based pharmacophore generation

The **1**-SUB1 and **2**-SUB1 complexes (PfSUB1, PvSUB1 and PkSUB1) were employed for structure-based (SB) pharmacophore generation by means of the e-Pharmacophore application (Maestro, version 9.2, Schrödinger, LLC, New York, NY, 2011). The outputs obtained by means of GOLD software were considered as the starting structures for SB pharmacophore generation. Compounds **1** and **2** were extracted from the enzymes and re-docked into the respective binding site using Glide software [37] applying rigid docking and using the score in place method to preserve the original conformation previously found. For this latter, Glide XP was used and the grid was generated with default settings using compound **1** as reference ligand. Subsequently, **1** and **2** were re-docked in the same conformation derived from the complex generated by GOLD software using Glide extra precision (XP) method by using the score in place method to preserve the original poses. Following this method, it was possible to generate a .Xpdes file containing the protein–ligand interaction data, necessary for developing a SB pharmacophore by means of e-Pharmacophore. The Glide pose was selected and used in e-Pharmacophore GUI. The ligand mode option was used to develop a pharmacophore hypothesis. The maximum feature option was set to 10, with a minimum inter-feature distance of 2.0 Å. Receptor-based excluded volumes were created using 0.5 as van der Waals scaling factor. Pharmacophore sites were automatically generated from the protein–ligand docked complex with Phase using the default set of six chemical features: hydrogen bond acceptor (A), hydrogen bond donor (D), hydrophobic (H), negative ionizable (N), positive ionizable (P), and aromatic ring (R) [38], no user-defined features were employed in this study. The e-Pharmacophore hypothesis was imported and managed into Phase according to docking studies. The obtained SB pharmacophores are shown in Fig. 6. The most conserved features among SB pharmacophores were clustered by Phase according to common features pharmacophore hypothesis generation. The resulting SB hypothesis (SUB1-PHA) consists of six features: three hydrogen-bond acceptors (A_1_, A_2_, and A_3_; represented by red vectors), one bond donor (D_2_; represented by light blue vectors), one hydrophobic function (H_1_; represented by a green sphere) and one negative ionizable centre (N_1_; represented by a dark red sphere). The inter features distance of AAADHN hypothesis is reported in Fig. 7. The fitness of all compounds was calculated by Phase by applying the SUB1-PHA model, employing excluded volumes, and using search for matches option. The conformers were generated by means of ConGen (ConfGen, version 2.3, Schrödinger, LLC, New York, 2011) during the “search for matches” calculations.

### 2.6 Pharmacophore validation

Database of Useful Decoys: Enhanced (DUD-E) web server (http://dude.docking.org access date May 2015) was used to generate a set of decoys starting from our active compounds **1**, **2** and other unique substrate-based SUB1 inhibitors for the selected *Plasmodium* species, represented by KS-182 (**3**) and KS-466 (**4**) and reported in literature [11] (Table S1). Peptidyl α-ketoamide **3** (Table S1) showed lower inhibitory potency against the PfSUB1, PkSUB1 and PvSUB1 (IC_50_ values: 6 μM, 6 μM and 12 μM, respectively) than compounds **1** and **2** (Fig. 1), while **4** (Table S1) showed an IC_50_ value comparable to **1** and **2** (PfSUB1, PkSUB1 and PvSUB1 IC_50_ values: 1 μM, 1 μM and 2 μM, respectively) [11]. It is worth noticing that these compounds were tested in the same condition of inhibitors **1** and **2**. For the active ligands DUD-E server provided 169 inactive ligands (redundant structures in the output files were delete) from a subset of the ZINC database (http://zinc.docking.org accessed date May 2015) filtered using the Lipinski rules for drug-likeness, for a total of 173 compounds between active and inactive (Table S1). Each of these inactive decoys is chosen to resemble the reference ligand physico-chemical properties but to have different 2D structure (e.g., very large difference of Tanimoto coefficient between active molecules and decoys). After the generation, the decoys sets were downloaded as four smiles files and imported into Maestro in order to perform a minimization by means of Macromodel (the same parameters reported for ligand preparation were applied). Furthermore, before submitting the obtained set of decoys to fitness evaluation, LigPrep application was used to prepare the ligands for removing potential erroneous structures and for assessing the stereochemistry of the active compounds. Notably, by visual inspection of selected decoys, we found that a relevant number of generated decoys (about 25%) maintained a significant peptidic character. A single file containing active molecules and decoys was created and submitted to Phase for evaluating the fitness against the SUB1-PHA. After decoys generation and pharmacophore fitness evaluation enrichment factor (EF) value, Güner and Henry score, i.e., goodness of hit-list (GH) were calculated by the Eqs. (1) and (2), respectively.

(1) $$\text{EF}=\frac{H_{a}/H_{t}}{\left(A/D\right)}$$

(2) $$\text{GH}=\left\{\frac{H_{a}\times (3A+Ht)}{4HtA}\right\}\times \left[1-\frac{\left(H_{t}-H_{a}\right)}{\left(D-A\right)}\right]$$

where *H*_t_ is the total number of molecules in the hit list found by pharmacophore screening (fitness cutoff 2.00), *H*_a_ represents the total active molecules found by screening applying a fitness cutoff value of 2.00, *A* is the total of the active molecules present in the database, while *D* is the total molecules present in the set. The range of GH score varies from 0 to 1. GH score 0 means a null model, while the GH score 1 means generation of an ideal model. Moreover, also the % yield of actives (%YA) and % ratio of actives (%RA) were evaluated by the Eqs. (3) and (4), respectively.

(3) $$\%\text{YA}=\left[\left(\frac{H_{a}}{H_{t}}\right)\times 100\right]$$

(4) $$\%\text{RA}=\left[\left(\frac{H_{a}}{A}\right)\times 100\right]$$

Results concerning the pharmacophore validation are provided in the Section 3 (Table 3) and in Table S1 in the Supplementary material.

### 2.7 SUB1 inhibition assays for IC_50_ determination

Inhibitory potency of compounds **1** and **2** against recombinant PfSUB1, PkSUB1 and PvSUB1 [11], [24] was assayed as previously described [5], [6], [11], using fluorogenic substrate SERA4st1F-6R12, which is peptide Ac-CKITAQDDEESC labelled on both cysteine side-chains with tetramethylrhodamine. The intact substrate displays low fluorescence due to non-covalent, concentration-dependent dimerization of the rhodamines. Cleavage within the peptide backbone allows dissociation of the rhodamine dimer and consequent fluorescence increase. One unit (1 U) of recombinant PfSUB1 is defined as the amount of protease that hydrolyses 1 pmol of SERA4st1F-6R12 in 1 min at a substrate concentration of 0.1 μM in digestion buffer (25 mM Tris-HCl pH 8.2, 12 mM CaCl_2_, 25 mM CHAPS) at 21 °C. For kinetic assays to determine IC_50_ values for test compounds, wells of a 96-well white microplate (Nunc) containing 48 μL purified enzyme (∼1 U/mL in digestion buffer), were supplemented in triplicate with 2 μL of various concentrations of the test compounds, freshly diluted in dry DMSO, prior to addition of 50 μL substrate (0.1 μM in digestion buffer). The resulting fluorescence increase was continuously monitored with time at 21 °C using a Cary Eclipse fluorescence spectrophotometer (Varian) equipped with a 96-well microplate reader accessory. Initial hydrolysis rates calculated from the resulting progress curves were plotted against test compound concentration to obtain IC_50_ values. Vehicle alone (DMSO) was used to obtain values for uninhibited enzyme activity and *para*-hydroxymercuribenzoate, a potent inhibitor of SUB1 [24], was used as a positive control inhibitor.

## 3 Results and discussion

**Fig. 2:**
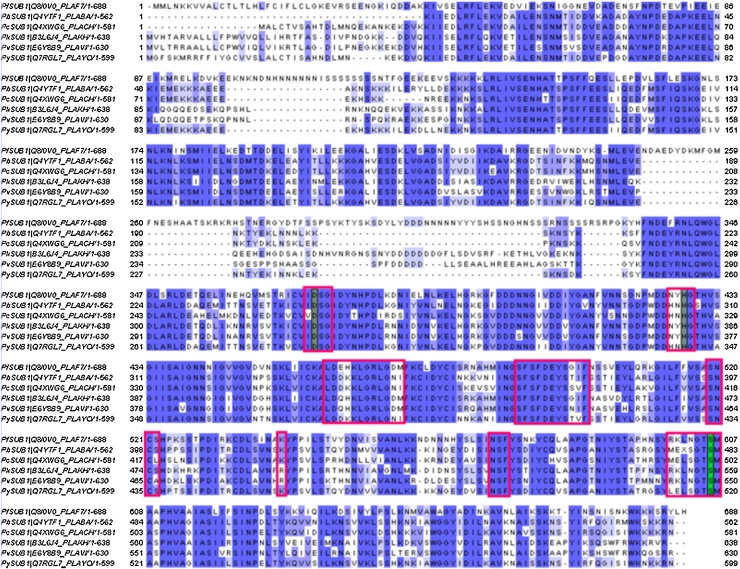
Multiple alignments between complete sequences of PfSUB1, PbSUB1, PcSUB1, PkSUB1, PvSUB1 and PySUB1. The intensity of the purple shading indicates the degree of conservation at specific residues. Catalytic triad residues are highlighted in each sequence. Residues included in the binding sites were rounded by a red line. The image was generated using jalview implemented in the EMBL-EBI website (https://www.ebi.ac.uk/). (For interpretation of the references to colour in this figure legend, the reader is referred to the web version of this article.)

Our computational investigation started by comparing the conserved regions of the catalytic domains of the SUB1 orthologues. To examine the degree of sequence homology within the binding core of the enzymes, the primary structures of all SUB1 proteases were obtained from the UniprotKB database and aligned with the PfSUB1 sequence previously investigated by us [21] (Fig. 2).

Although the alignment (Fig. 2 and Table 1) demonstrated a high degree of conservation of residues located around the catalytic triad, important differences in the binding sites of SUB1 orthologues were observed. We therefore generated, through homology modeling, the 3D structures of the catalytic cores of SUB1 orthologues for which the crystal structure is not available. Our original PfSUB1 homology model was developed by adopting a multiple template-based approach [11], [21], [26], [27], which allowed us to discover the most potent PfSUB1 inhibitors reported to date, namely **1** and **2** (Fig. 1) [21]. This methodology resulted in an improved quality of the final 3D-homology models [39], [40], [41]. For the present study, we adopted multiple template-based alignment technique using the PfSUB1 [24] (PDB code: 4LVN) and PvSUB1 [23] (PDB code: 4TR2) crystal structures as templates to build 3D models of the other SUB1 orthologues (see Section 2). As expected, the above-mentioned experimental structures showed a high degree of identity and similarity within the SUB1 orthologues (see homology modeling paragraph). The third top-ranking template (bacterial collagenolytic serine protease PDB code: 3VV3) had a dramatically lower degree of core identity (core region: PbSUB1 38%; PcSUB1 39%; PySUB1 38%; PkSUB1 37%; the percentage of identity was calculated by Prime [28] during template selection step).

**Fig. 3:**
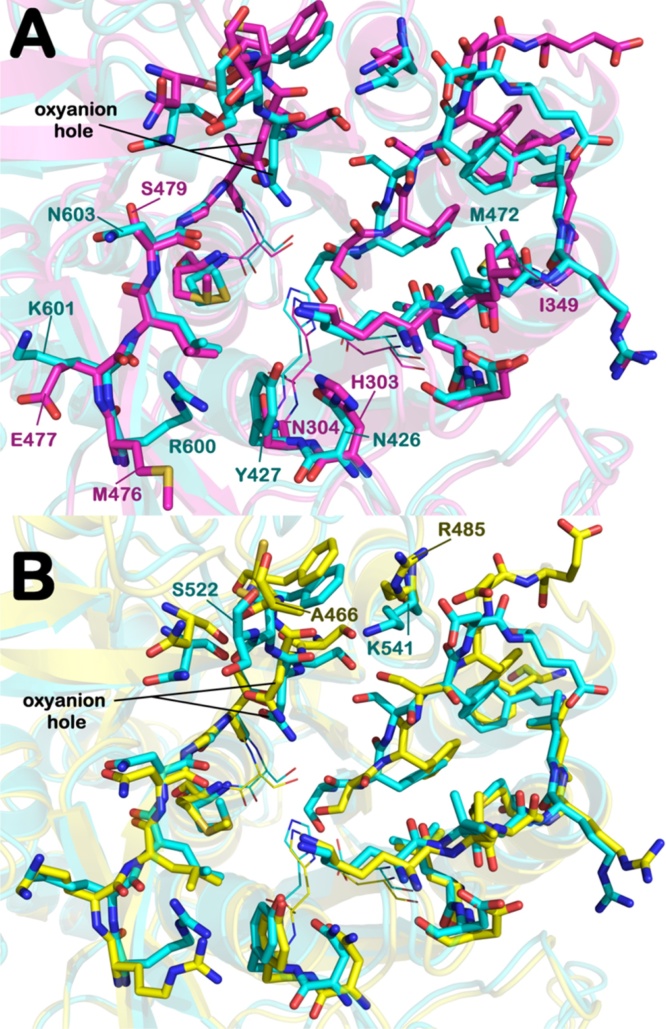
Superposition between the binding sites of PbSUB1 and PvSUB1 catalytic core with PfSUB1. A) Superposition between binding sites of the PbSUB1 homology model (magenta sticks) and the PfSUB1 crystal structure (cyan sticks) (PDB code 4LVN). Catalytic triad residues (His, Ser, Asp) are represented by lines. The numbering of PbSUB1 is in agreement with the sequence obtained from UniprotKB. B) Superposition between catalytic core of the PvSUB1 crystal structure (PDB code 4TR2) (yellow sticks) and the PfSUB1 crystal structure (PDB code 4LVN). Catalytic triad residues (His, Ser, Asp) are represented by lines. All hydrogens are removed for clarity. The picture was generated by PyMOL (The PyMOL Molecular Graphics System, v1.6-alpha; Schrodinger LLC, New York, NY, 2013). (For interpretation of the references to colour in this figure legend, the reader is referred to the web version of this article.)

By comparing the sequences and by superposing the 3D models of PbSUB1 (Fig. 3), PcSUB1 (Fig. S1) and PySUB1 (Fig. S2) on that of PfSUB1, we identified relevant differences in the putative active sites of these orthologues. In particular, as exemplified in Fig. 3A for PbSUB1, the most relevant differences from PfSUB1 are located in the S’ surface (replacement of PfSUB1 residues N603, K601, R600 by PbSUB1 residues S479, E477, M476, respectively), the S2 pocket (replacement of PfSUB1 residues Y427, N426 by N304, H303), and the S3 sub-site (PfSUB1 residue M472 replaced by I349 in PbSUB1). A similar result was obtained for PcSUB1 and PySUB1 when compared to PfSUB1 (Fig. S1 and S2, respectively).

In contrast, a comparison of PvSUB1 and PkSUB1 with PfSUB1 (Fig. 3B and Fig. S3, respectively) showed that the binding sites are superimposable, with PfSUB1 K541 and S522 residues replaced by R485 and A466 in PvSUB1, and only K541 replaced by R494 in PkSUB1. These amino acids replacements only marginally influence substrate binding, since they are located at the edge of the binding sites.

We next applied an intensive molecular docking protocol using the SUB1 crystal structures and homology models to investigate the binding modes of **1** and **2** (Fig. 1). Molecular docking was carried out using GOLD software, applying Goldscore as scoring function [35], [43] (see Section 2 for further details). Since inhibitors **1** and **2** were designed on the basis of a PfSUB1 endogenous substrate, we envisaged different recognition patterns of these inhibitors in complex with PbSUB1, PcSUB1 and PySUB1, while we expected similar binding modes for **1** and **2** when docked into PvSUB1 and PkSUB1.

**Fig. 4:**
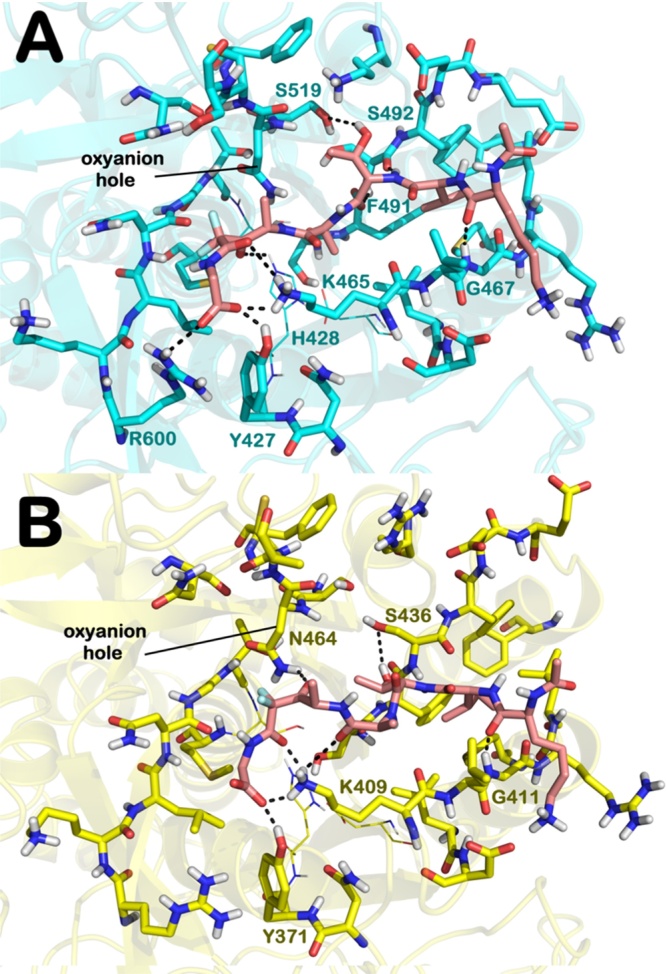
Docking of **1** in PfSUB1 and PvSUB1. A) Docked pose of **1** (pink sticks) into the PfSUB1 crystal structure binding site (PDB code 4LVN) [21]. B) Docked pose of **1** (pink sticks) into the PvSUB1 binding site (PDB code 4TR2) (yellow sticks). Key residues are represented by sticks, while the catalytic triad is represented by lines (H428, S606, and D372 for PfSUB1; H372, S549, and D316 for PvSUB1). Non-polar hydrogens are removed for clarity. H-bonds are represented by black dotted lines. (For interpretation of the references to colour in this figure legend, the reader is referred to the web version of this article.)

As previously described [21], the output of docking calculations reveals that compound **1** in complex with PfSUB1 (Fig. 4A) is engaged in H-bonding with S492, S519, G467, and H428, while K465 is able to form a H-bond with the carbonyl group of the difluorostatone amide. The free acidic functionality of **1** establishes a series of polar contacts with R600, Y427 and K465. The formation of these polar contacts, along with the favorable binding conformation of **1**, allows the difluorostatone electrophilic carbon of the carbonyl group (described in [21]) to lie in the proximity of the catalytic S606 (5.0 Å) and accounts for its high binding score (93.56). The calculated values highlighted the high affinity of **1** for PfSUB1. A similar output was found for inhibitor **2** (Fig. S4). Analogously, due to the similarities in SUB1 binding sites, **1** and **2** are expected to bind tightly to PvSUB1 and PkSUB1.

The molecular docking calculation of **1** into the PvSUB1 binding site (Fig. 4B) is similar to that found for **1** in PfSUB1 (docking of **2** into PvSUB1 is reported in Fig. S5). Compound **1** occupies the full length of the cleft and strongly interacts with both the S′ and S2 regions through its P1′ and P1 moieties, respectively (Fig. 4B).

In particular, a network of H-bonds with Y371 and K409 is formed, analogous to that observed in the PfSUB1 structure. The targeted residue K409 (K465 in PfSUB1) appears critical for enzyme inhibition [24]. Importantly, the electrophilic carbon of the difluorostatone carbonyl group lies 5.7 Å from the catalytic S549. In addition compound **1** appeared able to directly interact with oxyanion hole by H-bonding with N464. Further contacts were detected with a central residue of the S1 pocket (S436). Notably, the P4 residue of **1** is accommodated into the hydrophobic S4 region as found for PfSUB1, allowing contacts with G411. The Goldscore of **1** in PvSUB1 (84.89) is slightly lower than that of **1** in PfSUB1 (93.56). Based on this data we expect that **1** should be able to inhibit PvSUB1 with a potency similar to PfSUB1. A similar output was also found for **1** when docked into PkSUB1 (Fig. S6). The results for **2** in PkSUB1 are provided in the Supplementary material (Fig. S7).

Gratifyingly, the predicted affinities of **1** and **2** for PvSUB1 and PkSUB1 were supported by experimental data (Table 2). In fact, both inhibitors **1** and **2** showed comparable inhibitory potencies when tested against the target enzymes, confirming that, concerning clinically relevant *Plasmodium* species, **1** and **2** are pan-SUB1 inhibitors.

**Fig. 5:**
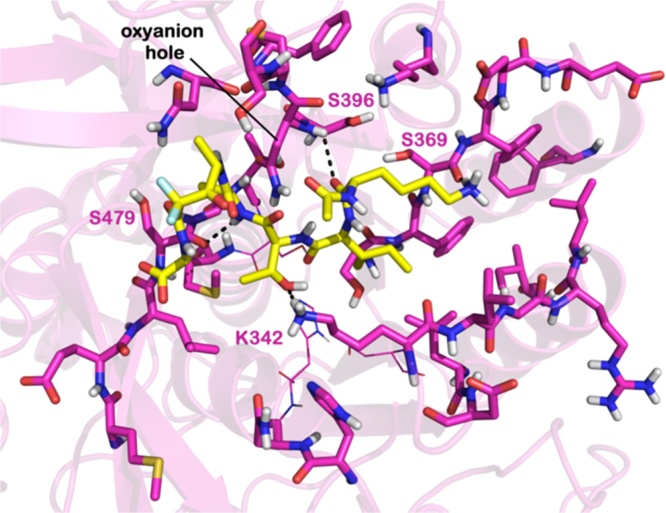
Docked pose of **1** (yellow sticks) into the PbSUB1 homology model binding site (magenta sticks). Key residues are represented by sticks, while the catalytic triad is represented by lines (H305, S482, and D249). Non-polar hydrogens were removed for clarity. H-bonds are represented by black dotted lines. (For interpretation of the references to colour in this figure legend, the reader is referred to the web version of this article.)

On the other hand, significant dissimilarities were found among the predicted binding modes of **1** and **2** when docked into PbSUB1, PcSUB1 and PySUB1 in comparison with the binding modes in PfSUB1, PvSUB1 and PkSUB1. The docking of **1** into the PbSUB1 binding site is shown in Fig. 5, while the other docking results, namely **2** with PbSUB1 (Fig. S8), **1** with PcSUB1 and PySUB1 (Fig. S9 and S10, respectively) and **2** with PcSUB1 and PySUB1 (Figs. S11 and S12, respectively) are presented in the Supplementary material.

The docking calculation for **1** into the PbSUB1 binding site (Fig. 5) revealed that **1** is unable to correctly interact with the catalytic triad. In particular, the distance of the difluorostatone electrophilic carbonyl carbon from the catalytic Ser (S482) is 11.8 Å (compared to 5.0 Å from S606 in the PfSUB1 binding site).

Moreover, **1** establishes a smaller number of contacts with PbSUB1, forming only three H-bonds with K372, S479 and S369. Due to the differences between PfSUB1 and PbSUB1 in the amino acid composition of the S1′ and S4 sub-sites, **1** does not project into the PbSUB1 S4 region nor into the S1′ sub-site, so its carboxylic acid moiety cannot create the key contacts observed for PfSUB1. Based on the observed binding mode, we can predict a lower inhibitory potency for **1** against all three murine malaria parasite SUB1 orthologues. Accordingly, the Goldscore found for **1** in PbSUB1 is substantially lower (67.19) than that for **1** in PfSUB1 (93.56). These predictions are in agreement with the experimental data on PbSUB1 obtained for other PfSUB1 inhibitors designed on the basis of the endogenous substrate [11]. The analogous results for **2** in PbSUB1 are presented in Fig. S8. Similar bindings have been predicted for **1** in PcSUB1 and PySUB1 (the GoldScore of **1** in PcSUB1 is 67.81 and in PySUB1 is 71.70) and are provided in Fig. S9 and S10, respectively (results for compound **2** in PcSUB1 and PySUB1 are reported in Fig. S11 and S12, respectively). These findings fully support the key role of the S′ region in SUB1-ligand recognition in the human malaria pathogen enzymes.

Based on the docking analysis of **1** and **2** in complex with PfSUB1, PvSUB1 and PkSUB1, we developed a comprehensive pharmacophore useful for the rational design of pan-SUB1 inhibitors. For generating a multiple SB pharmacophore, we took into account our knowledge in pharmacophore modeling by using Phase [27], [44], [45], [46], [47], and the e-Pharmacophore protocol [27]. This procedure combines pharmacophore perception with protein ligand energetic terms computed by the Glide XP (extra precision) scoring function (see Experimental section for details) that improves the reliability of the 3D pharmacophores.

**Fig. 6:**
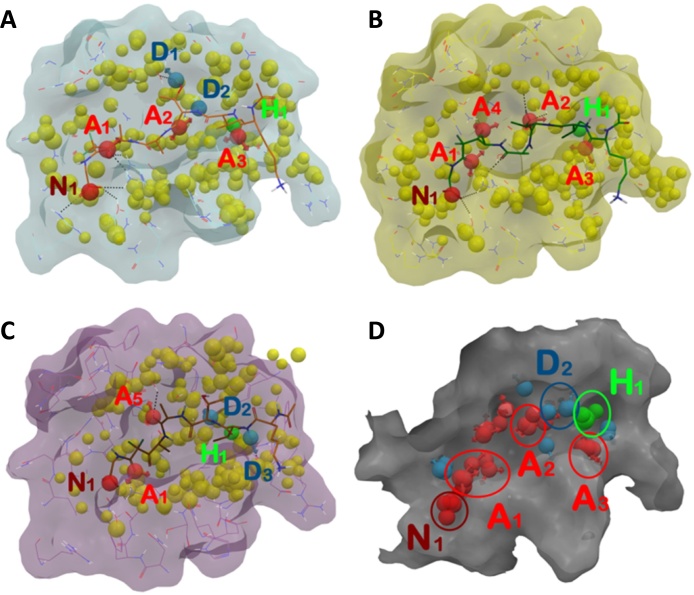
Features of the SB-pharmacophores and their superposition. (A–C) SB pharmacophores obtained for PfSUB1, PvSUB1 and PkSUB1, respectively with compound **1**. (D) Conserved features among the six SB pharmacophores highlighted by color-coded circles. The yellow spheres represent the excluded volumes. H-bonds are represented by black dotted lines. Pictures were generated by means of Maestro. (For interpretation of the references to colour in this figure legend, the reader is referred to the web version of this article.)

**Fig. 7:**
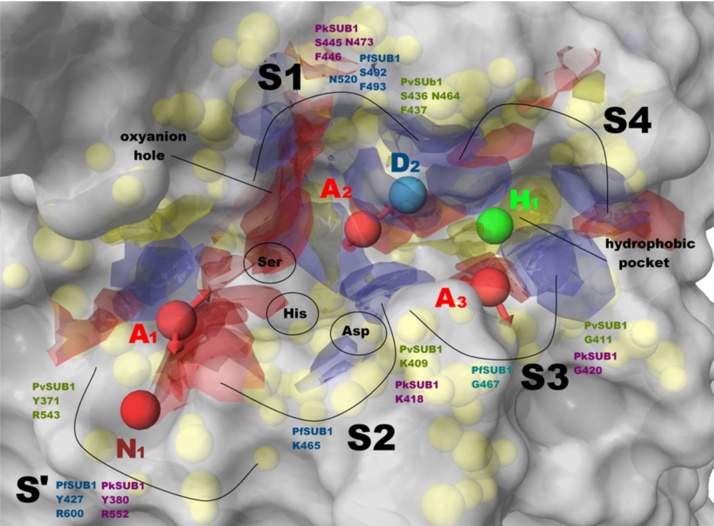
SUB1-PHA (inter features distance: N_1_–A_1_ 4.10 Å; A_1_–A_2_ 9.17 Å; N_1_-D_2_ 13.91 Å; A_2_–A_3_ 6.50 Å; A_2_–H_1_ 5.61 Å; D_2_–A_3_ 5.25 Å; D_2_–H_1_ 4.19 Å; A_3_–H_1_ 4.12 Å; N_1_–A_1_–A_2_ angle 123.1°) implemented with SUB1 recognition sites (S′-S4) highlighting the conserved target residues for all the enzymes taken into account coupled to SiteMap outputs (the maps of binding sites for a potential ligand interaction are represented as a solid surface: red = acceptor; blue = donor; yellow = hydrophobic). The yellow spheres represent the comprehensive excluded volume. The catalytic triad (Ser-His-Asp) is depicted based on the superposition of PfSUB1, PvSUB1 and PkSUB1. The image was generated by means of Maestro.

The poses obtained by the classical docking calculations described in the previous paragraphs were used to generate a SB pharmacophore for each SUB1 orthologue through the e-Pharmacophore application. Superposition between **1** and SB pharmacophore models is shown in Fig. 6A–C (PfSUB1, PvSUB1 and PkSUB1 respectively); for compound **2**, pharmacophore modeling studies are provided in Fig. S13. The multiple SB pharmacophores were then analyzed and the common features were clustered in order to obtain a comprehensive pharmacophore model (Fig. 6D). This model takes into account the information derived from the retrieved binding modes coupled to binding energetic terms. The model was further elaborated by employing the SiteMap output depicted in Fig. 7. This model represents the first comprehensive SUB1-pharmacophore (SUB1-PHA) containing all the necessary features that a molecule must possess in order to inhibit all three SUB1 orthologues under consideration.

As reported in Fig. 7, the SUB1-PHA consists of six features: three hydrogen-bond acceptors (A_1_, A_2_, and A_3_; represented by red vectors), one hydrogen-bond donor (D_2_; represented by light blue vectors), one hydrophobic function (H_1_; represented by a green sphere) and one negatively ionizable centre (N_1_; represented by a dark red sphere). In particular, features N_1_ and A_1_ are essential for interacting through the polar S' and S2 sub-pockets with conserved Arg, Tyr and Lys residues respectively. The A_2_ and D_2_ features are essential for establishing polar contacts with the central recognition site (S1) with conserved Ser, Phe (backbone) as well as with Asn (oxyanion hole) residues. Feature A_3_ was found to be important for interacting at the S3 site with a conserved Gly. Moreover, the hydrophobic function H_1_ is required to interact at the S4 in the conserved hydrophobic sub-pocket formed by three Phe residues and a Leu as already highlighted by us ^21^. SiteMap outputs provide information about the polar/hydrophobic requirements for the linker connecting the selected features.

**Fig. 8:**
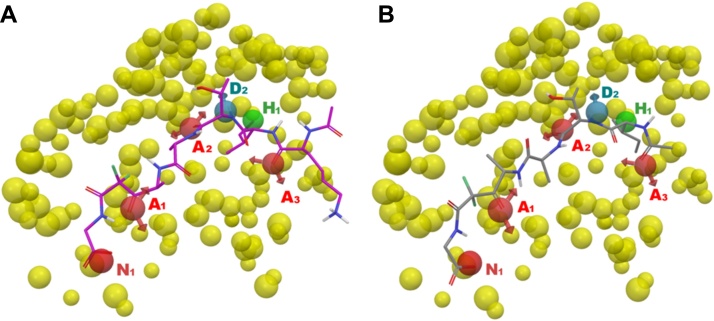
Superposition among SUB1-PHA and **1** (panel A) and **2** (Panel B). The pictures were generated by means of Maestro.

Next, we performed a preliminary in silico validation of our SUB1-PHA using a pharmacophore validation method based on the generation of decoys set, a procedure largely used to assess the ability of pharmacophore models to discriminate between active or inactive molecules [48], [49], [50], [51], [52], [53], [54], [55], [56], [57], [58]. Starting from compounds **1** and **2** (superposed to the SUB1-PHA in Fig. 8), and literature compounds **3** and **4** (KS-182 and KS-466, respectively) [59] we generated 169 decoys by means of DUD-E server [60], [61] (see Experimental Section for further details about the selection of active compounds). So, the database consists of 173 compounds (*D*) including 4 known active molecules (*A*) (Table 3). The database screening results (Table 3 and Table S1) showed that 5 molecules were found as hits (*H*_t_) applying a fitness cutoff value of 2.00 (this value could represent the lower limit to consider a molecule as active). Among these, 3 compounds (*H*_a_) belong to the four known inhibitors (**1,2** and **4**, the only compounds able to match all the pharmacophore sites).

Compound **3** was ranked 9th, matching five SUB1-PHA sites. Concerning the fitness estimation of decoys set (Table S1), it is well evident the inability for the inactive compounds to match all the features of SUB1-PHA as highlighted by small values of fitness (all decoys matched 3–5 features, but none of them matched all SUB1-PHA features). It is also worth noticing that around 25% of decoys presented a peptidic character. The EF was calculated to be 25.95, which means that is 25.95 times more probable to identify active compounds from databases than expected by chance. The calculated GH score value of 0.63, greater than 0.5, indicates a good reliability of the model.

Despite the small number of molecules so far described as SUB1 inhibitors, the results of this preliminary in silico analysis suggest that the SUB1-PHA model could be successfully employed for virtual screening to find novel SUB1s inhibitors.

## 4 Conclusion

We have herein presented the first comprehensive approach for rationalizing the structural requirements for inhibition of SUB1 from different *Plasmodium* species. We built up homology models of SUB1 from different *Plasmodium* species adopting multiple template-based alignments. The available X-ray structures and our homology models were used for Molecular Docking studies using the potent PfSUB1 inhibitors **1** and **2** previously developed by us for our inclusive computational analysis. Computational studies were validated by in vitro testing, indicating our compounds as the most potent pan-SUB1 inhibitors reported to date. On the basis of the rational approach described here, we have predicted and experimentally demonstrated that inhibitors **1** and **2** are able to potently inhibit SUB1 enzymes of the most important human malaria pathogens, confirming that the development of pan-SUB1 inhibitors is a feasible task [11]. We also developed and validated a comprehensive pharmacophore model (SUB1-PHA), and we are confident that it will be useful for the rational design of optimized pan-inhibitors by adopting fragment-based techniques and/or for a classical virtual screening campaign. Taken together, these findings pave the way to the development of a novel class of pan-antimalarial agents for human infections by targeting serine protease SUB1. It is also worth noting that, based on the studies reported here, compounds designed on the basis of endogenous substrates of PfSUB1 (such as **1** and **2**) would not be expected to possess similar activity against *P. berghei*, *P. chabaudi* or *P. yoelii*. As a consequence, suitable animal models of the disease should be developed in the future to investigate the in vivo antimalarial potential of substrate-based inhibitors of SUB1 and for progressing them into the drug discovery pipeline.

## Figures and Tables

**Table 1:** Sequence comparison of PbSUB1 (UniprotKB code: Q4YVE1; 562 aa), PySUB1(UniprotKB code: Q7RGL7; 599 aa), PcSUb1 (UniprotKB code: Q4XWG6; 581 aa), PvSUB1 (UniprotKB code: E6Y8B9; 630 aa), and PkSUB1 (UniprotKB code: B3L6J4; 638 aa) with PfSUB1 (UniprotKB code: Q8I0V0; 688 aa).

SUB1s	Sequence identity^a^	Identical residues^a^	Similar residues^a^	Core identity^b^
PbSUB1	49.2%	339	156	64%
PySUB1	49.7%	345	172	64%
PcSUB1	48.5%	334	181	67%
PvSUB1	55.0%	380	161	74%
PkSUB1	55.3%	382	164	75%

a The calculation was performed by Clustal omega provided by the Uniprot website (http://www.uniprot.org/align).

b The core of PfSUB1 lies between F338 and K669 as determined from the crystal structure [42]. The calculation was performed by ClustalW implemented in Prime 3.0.

**Table 2:** IC_50_ (μM) of SUB1 inhibitors **1** and **2**.

Compound	PfSUB1^a^	PkSUB1	PvSUB1
**1**	0.60	1.12	2.5
**2**	0.60	0.68	2.2

a IC_50_ values as presented in Ref. [3].

**Table 3:** EF and GH scores obtained by the application of SUB1-PHA in a database screening.

Parameters	Values
Total molecules in database (*D*)	173
Total number of actives in database (*A*)	4
Total hits (*H*_t_)	5
Active hits (*H*_a_)	3
% Yield of actives (YA)	0.60
% Ratio of actives (RA)	0.75
Enrichment factor (EF)	25.95
Goodness of hit score (GH)	0.63
