# Supplementary material for: In silico study of subtilisin-like protease 1 (SUB1) from different Plasmodium species in complex with peptidyl-difluorostatones and characterization of potent pan-SUB1 inhibitors
Source: J Mol Graph Model. 2016 Mar;64:121–30. doi: 10.1016/j.jmgm.2016.01.005 (PMC5276822; doi:10.1016/j.jmgm.2016.01.005)
Supplement: Supplementary file 1 [file mmc1.docx]

SUPPLEMETARY MATERIAL

**In silico study of subtilisin-like protease 1 (SUB1) from different *Plasmodium* species in complex with peptidyl-difluorostatones and characterization of** **potent pan-SUB1 inhibitors**

Simone Brogi,^a,b,c^ Simone Giovani,^a,b,c^ Margherita Brindisi,^a,b,c^ Sandra Gemma,*^,a,b,c^ Ettore Novellino,^a,d^ Giuseppe Campiani,*^,a,b,c^ Michael J. Blackman,^e^ Stefania Butini ^a,b,c^

*^a^European Research Centre for Drug Discovery and Development (NatSynDrugs), University of Siena, via Aldo Moro 2, 53100, Siena, Italy*

*^b^Dipartimento di Biotecnologie, Chimica e Farmacia, University of Siena, via Aldo Moro 2, 53100, Siena, Italy*

*^c^Centro Interuniversitario di Ricerche sulla Malaria (CIRM), University of Perugia, Perugia, Italy*

*^d^Dipartimento di Farmacia, University of Naples Federico II, Via D. Montesano 49, 80131, Naples, Italy*

*^e^Division of Parasitology, MRC National Institute for Medical Research, Mill Hill, London NW7 1AA, UK*

* Corresponding author. Tel.: +39 0577 234326; fax: +39 0577 234254; e-mail: [gemma@unisi.it](mailto:gemma@unisi.it)

* Corresponding author. Tel.: +39 0577 234172; fax: +39 0577 234254; e-mail: [campiani@unisi.it](mailto:campiani@unisi.it)

**Table of Contents**

**Figure S1-S13** S2-S14

**Table S1** S15


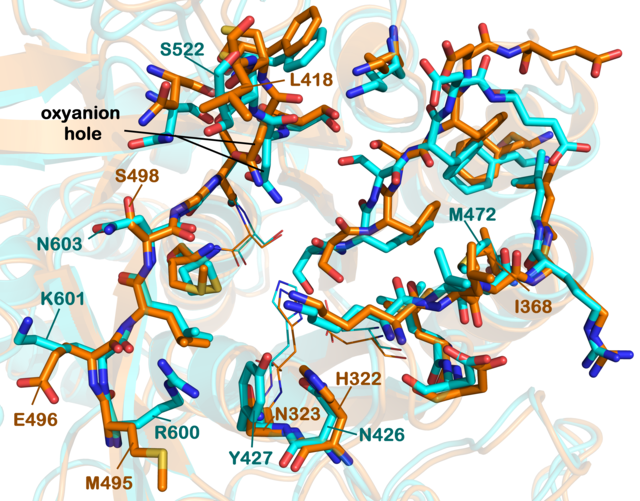


**Figure S1.** Superposition between PcSUB1 homology model (orange sticks) and PfSUB1 crystal structure (cyan sticks) binding sites. The catalytic triad (His, Ser, Asp) is represented by lines. All the hydrogens were removed for the sake of clarity. The picture were generated by PyMOL (The PyMOL Molecular Graphics System, v1.6-alpha; Schrodinger LLC, New York, NY, 2013).

**
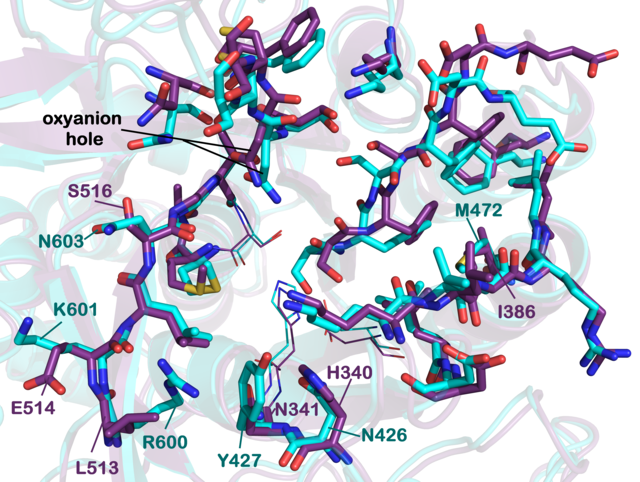
**

**Figure S2.** Superposition between PySUB1 homology model (purple sticks) and PfSUB1 crystal structure (cyan sticks) binding sites. The catalytic triad (His, Ser, Asp) is represented by lines. All the hydrogens were removed for the sake of clarity.

**
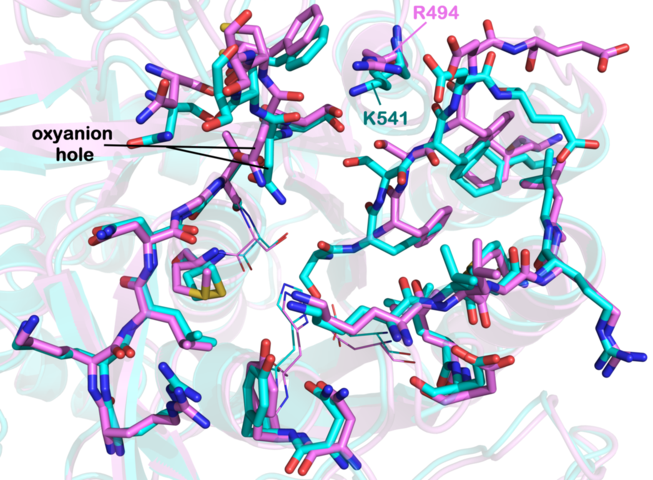
**

**Figure S3.** Superposition between PkSUB1 homology model (pink sticks) and PfSUB1 crystal structure (cyan sticks) binding sites. The catalytic triad (His, Ser, Asp) is represented by lines. All the hydrogens were removed for the sake of clarity.


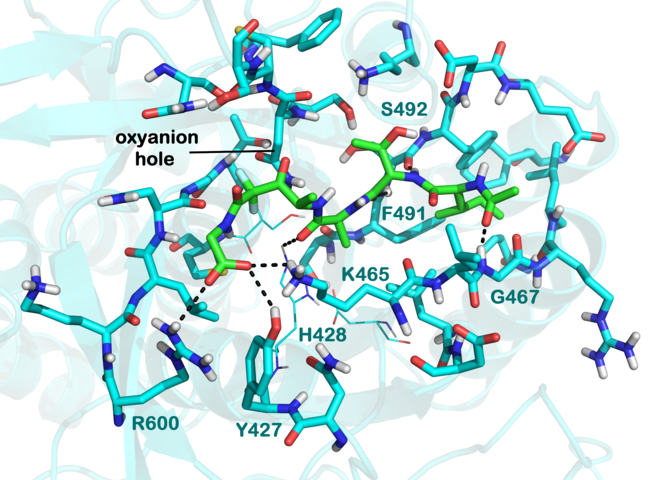


**Figure S4.** Docked pose of **2** (green sticks) into PfSUB1 crystal structure (PDB code 4LVN) (cyan sticks) binding site. Key residues are represented by sticks, while the catalytic triad is represented by lines. **2** showed an inhibitory potency similar to that of **1** (IC_50_ of **2 =** 0.6 μM), in agreement with its docking output, showing a similar pattern of interaction with respect to **1** (H-bonds with R600, Y427, K465, H428, S492, and G467) and also a comparable Goldscore (88.19). Non-polar hydrogens were removed for the sake of clarity. H-bonds are represented by black dotted lines.


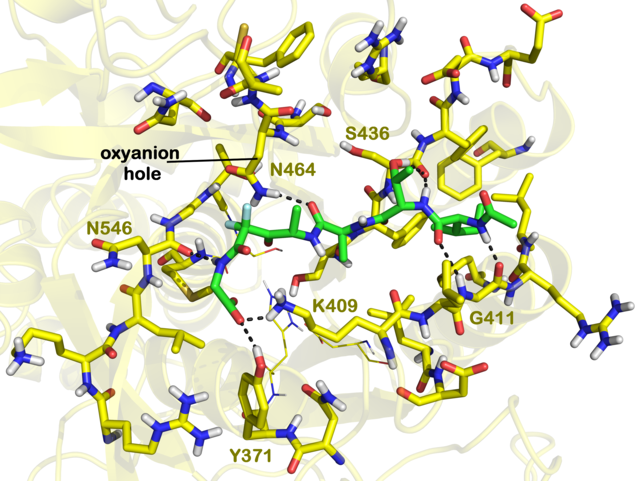


**Figure S5.** Docked pose of **2** (green sticks) into PvSUB1 binding site (PDB code 4TR2) (yellow sticks). Key residues are represented by sticks, while the catalytic triad is represented by lines. Non-polar hydrogens were removed for the sake of clarity. The calculated values of Goldscore is 82. The distance between electrophilic carbon of **2** and oxygen from S549 is 6.0 Å. H-bonds are represented by black dotted lines.

**
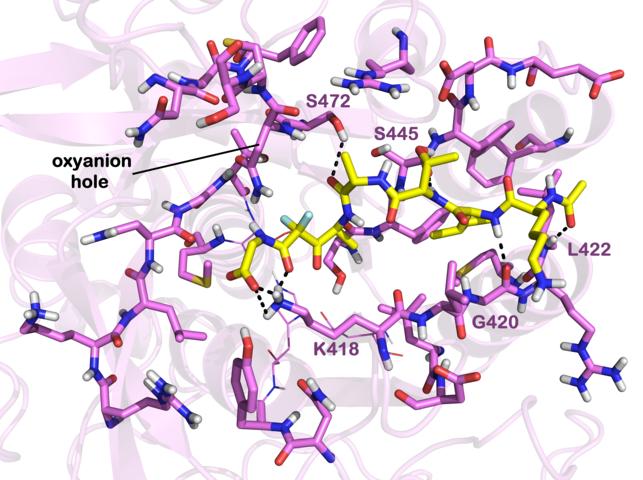
**

**Figure S6.** Docked pose of **1** (yellow sticks) into PkSUB1 homology model (pink sticks) binding site. Key residues are represented by sticks, while the catalytic triad is represented by lines (H381, S558, and D325). Non-polar hydrogens were removed for the sake of clarity. The calculated values of Goldscore is 86.13. The distance between electrophilic carbon of **1** and oxygen from S558 is 4.19 Å. H-bonds are represented by black dotted lines.

**
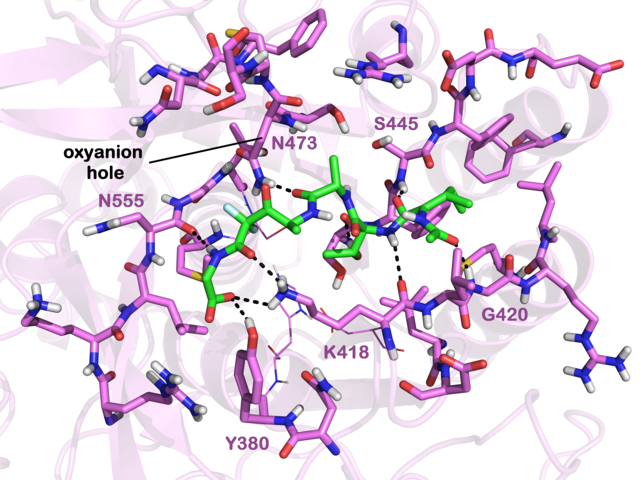
**

**Figure S7.** Docked pose of **2** (green sticks) into PkSUB1 homology model (pink sticks) binding site. Key residues are represented by sticks, while the catalytic triad is represented by lines. Non-polar hydrogens were removed for the sake of clarity. The calculated values of Goldscore is 91.01. The distance between electrophilic carbon of **2** and oxygen from S558 is 6.1 Å. H-bonds are represented by black dotted lines.

**
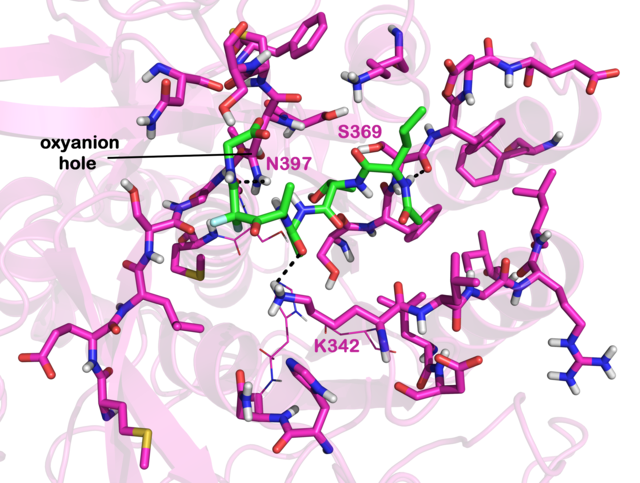
**

**Figure S8**. Docked pose of **2** (green sticks) into PbSUB1 homology model binding site (magenta sticks). Key residues are represented by sticks, while the catalytic triad is represented by lines. Non-polar hydrogens were removed for the sake of clarity. The calculated values of Goldscore is 66. The distance between electrophilic carbon of **2** and oxygen from S482 is 9.1 Å. H-bonds are represented by black dotted lines.


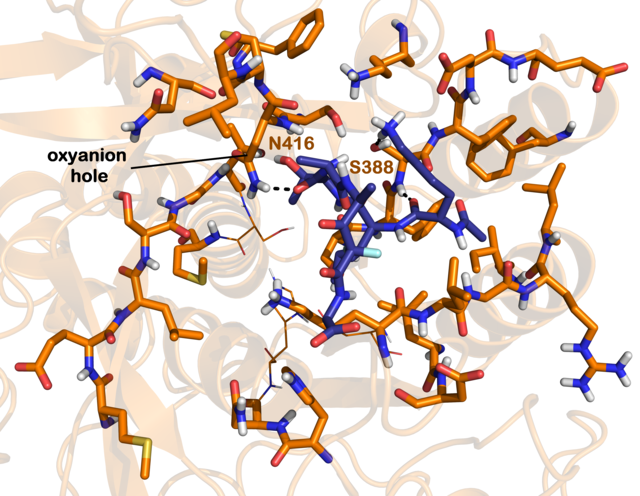


**Figure S9**. Docked pose of **1** (dark purple sticks) into PcSUB1 homology model (orange sticks) binding site. Key residues are represented by sticks, while the catalytic triad is represented by lines (H324, S501, and D268). Non-polar hydrogens were removed for the sake of clarity. The calculated values of Goldscore is 67.81. The distance between electrophilic carbon of **1** and oxygen from S501 is 10.6 Å. H-bonds are represented by black dotted lines.

**
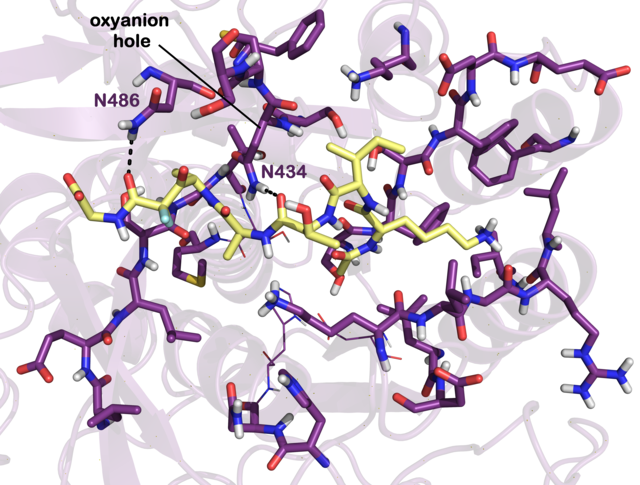
**

**Figure S10**. Docked pose of **1** (light yellow sticks) into PySUB1 homology model (purple sticks) binding site. Key residues are represented by sticks, while the catalytic triad is represented by lines (H342, S519, and D286). Non-polar hydrogens were removed for the sake of clarity. The calculated values of Goldscore is 71.70. The distance between electrophilic carbon of **1** and oxygen from S519 is 9.8 Å. H-bonds are represented by black dotted lines.

**
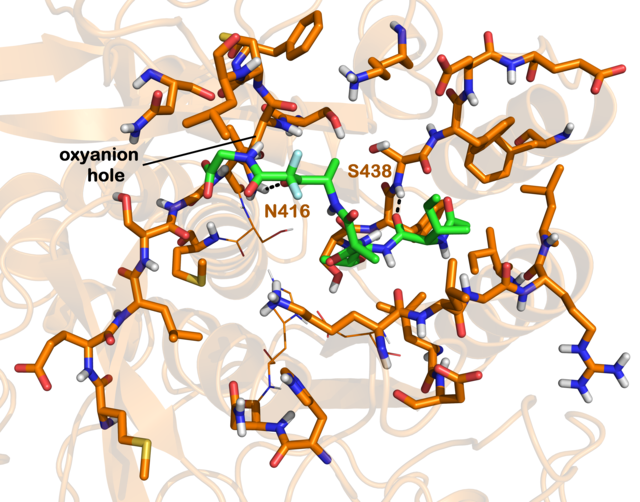
**

**Figure S11**. Docked pose of **2** (green sticks) into PcSUB1 homology model (orange sticks) binding site. Key residues are represented by sticks, while the catalytic triad is represented by lines. Non-polar hydrogens were removed for the sake of clarity. The calculated values of Goldscore is 65.08. The distance between electrophilic carbon of **2** and oxygen from S501 is 8.5 Å. H-bonds are represented by black dotted lines.

**
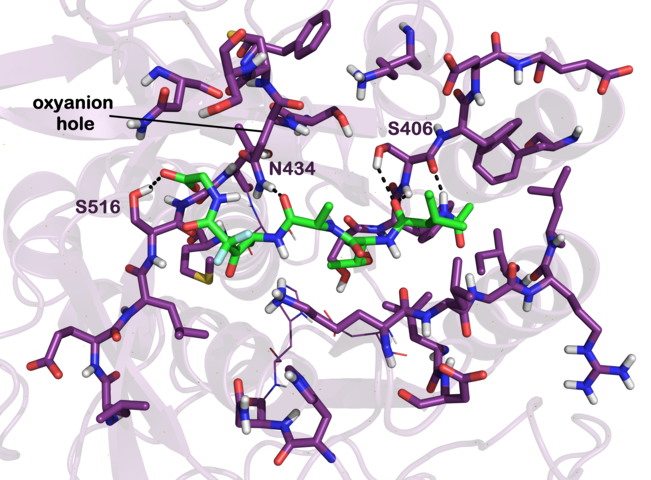
**

**Figure S12**. Docked pose of **2** (green sticks) into PySUB1 homology model (purple sticks) binding site. Key residues are represented by sticks, while the catalytic triad is represented by lines. Non-polar hydrogens were removed for the sake of clarity. The calculated values of Goldscore is 66.24. The distance between electrophilic carbon of **2** and oxygen from S519 is 7.6 Å. H-bonds are represented by black dotted lines.

**
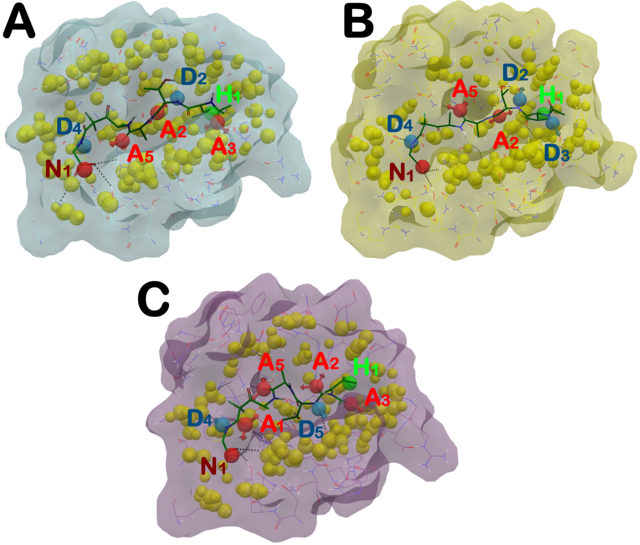
**

**Figure S13.** (A-C) SB pharmacophore obtained for PfSUB1, PvSUB1 and PkSUB1, respectively with compound **2**. The yellow spheres represent the excluded volume. H-bonds were represented by black dotted lines. Pictures were generated with Maestro.

**Table S1.** Evaluation of fitness for reference compounds **1**-**4** and for the generated decoys set **(**169 compounds) as calculated by applying SUB1-PHA using Phase software. Codes after the smiles notation are referred to the decoys generation performed by means of DUD-E server.

| **Rank** | **Compound** | **Fitness** |
| --- | --- | --- |
| 1 | **(1**) CC[C@H](C)[C@@H](C(=O)N[C@@H]([C@@H](C)O)C(=O)N[C@@H](C)C(=O)N[C@@H](C)C(=O)C(C(=O)NCC(=O)  [O-])(F)F)NC(=O)[C@H](CCCC[NH3+])NC(=O)C  Cpd1 P10000001 | 2.742 |
| 2 | (**2**) CC[C@H](C)[C@@H](C(=O)N[C@@H]([C@@H](C)O)C(=O)N[C@@H](C)C(=O)N[C@@H](C)C(=O)C(C(=O)NCC(=O)[O-])(F)F)NC(=O)C  Cpd2 P10000002 | 2.510 |
| 3 | c1cc(ccc1C[C@@H](C(=O)N[C@@H](CSSC[C@H](C(=O)N)NC(=O)[C@@H](Cc2ccc(cc2)O)N)C(=O)N)[NH3+])O  C71788315 P111270361 | 2.437 |
| 4 | c1ccc(c(c1)C(=O)N[C@@H](CO)C(=O)N[C@@H](CC(=O)O)C(=O)N[C@@H](CCCCNc2ccc(cc2[N+](=O)[O-])[N+](=O)[O-])C(=O)N3CCC[C@H]3C(=O)[O-])N  C98224537 P156217441 | 2.371 |
| 5 | (**4**) CC[C@@H](C)[C@H](C(=O)N[C@H]([C@@H](C)O)C(=O)N[C@H](C)C(=O)N[C@H](C)C(=O)C(=O)NCCC(=O)[O])NC(=O)[C@@H](CCCC  [NH3+])NC(=O)C  KS466 P10000004  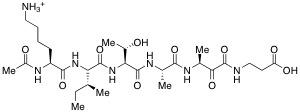 | 2.368 |
| 6 | C(C[NH3+])CO[P@@](=O)([O-])O[C@@H]1[C@H]([C@@H]([C@H]([C@@H]([C@@H]1O)O[P@@](=O)(O)[O-])OP(=O)([O-])[O-])O)O  C31544734 P151967373 | 1.739 |
| 7 | c1c(c(c(c[n+]1CCCC[C@@H](C(=O)[O-])N)CC[C@@H](C(=O)[O-])N)CCC[C@@H](C(=O)[O-])N)CC[C@@H](C(=O)[O-])[NH3+]  C35024527 P90243612 | 1.716 |
| 8 | CC(C)[C@@H](C(=O)OCCOCn1cnc2c1nc([nH]c2=O)NCNc3[nH]c(=O)c4c(n3)n(cn4)COCCOC(=O)[C@H](C(C)C)N)[NH3+]  C72130492 P111975713 | 1.705 |
| 9 | (**3**) CC[C@@H](C)[C@H](C(=O)N[C@H]([C@@H](C)O)C(=O)N[C@H](C)C(=O)N[C@H](CC)C(=O)C(=O)NC1CC1)NC(=O)[C@@H](CCCC  [NH3+])NC(=O)C  KS182 P10000003  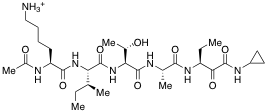 | 1.702 |
| 10 | c1c(c[n+](c(c1CC[C@@H](C(=O)[O-])N)CCC[C@@H](C(=O)[O-])N)CCCC[C@@H](C(=O)[O-])[NH3+])CC[C@@H](C(=O)[O-])N  C35024788 P51987324 | 1.694 |
| 11 | COC(=O)CNC(=O)[C@H](CSSC[C@@H](C(=O)NCC(=O)OC)NC(=O)CC[C@@H](C(=O)OC)[NH3+])NC(=O)CC[C@@H](C(=O)OC)[NH3+]  C77293156 P120588974 | 1.597 |
| 12 | C1C[NH+](CCN(CCC[NH+](CCN(C1)CC[P@](=O)(O)[O-])CCP(=O)([O-])[O-])CC[P@](=O)(O)[O-])CC[P@](=O)(O)[O-]  C68604313 P105279351 | 1.572 |
| 13 | c1cc(ccc1C[NH+](C[P@@](=O)(O)[O-])C[P@](=O)(O)[O-])CN(C[P@](=O)(O)[O-])C[P@](=O)(O)[O-]  C19900847 P105475150 | 1.539 |
| 14 | CN(Cc1cnc2c(n1)c(nc(n2)N)N)c3ccc(cc3)C(=O)N[C@H](CCC(=O)NCCNC(=O)CCCC[C@H]4[C@@H]5[C@H](CS4)NC(=O)N5)C(=O)[O-]  C72131520 P111978508 | 1.468 |
| 15 | CN(Cc1cnc2c(n1)c(nc(n2)N)N)c3ccc(cc3)C(=O)N[C@@H](CCC(=O)NCCNC(=O)CCCC[C@H]4[C@@H]5[C@H](CS4)NC(=O)N5)C(=O)[O-]  C72131519 P111978507 | 1.468 |
| 16 | Cc1cc(cc(c1O)C[NH+](CC(=O)[O-])CC(=O)[O-])C2(c3ccccc3S(=O)(=O)O2)c4cc(c(c(c4)C[NH+](CC(=O)[O-])CC(=O)[O-])O)C  C76945794 P120041488 | 1.411 |
| 17 | C[C@@H]1[C@@H]([C@@H]([C@H]([C@H](O1)OC[C@@H]2[C@H]([C@@H]([C@H]([C@@H](O2)OCCCCCCCCOC(=O)C)NC(=O)C)O)O[C@H]3[C@@H]([C@H]([C@@H]([C@H](O3)CO)O)O)NC(=O)C)O)O)O  C94307100 P147807931 | 1.378 |
| 18 | c1ccc(cc1)CC[C@@H](C(=O)NCCCC[C@@H](C(=O)N2CCC[C@@H]2C(=O)[O-])[NH2+][C@@H](CCc3ccccc3)C(=O)[O-])[NH2+][C@@H](CCCC[NH3+])C(=O)N4CCC[C@@H]4C(=O)[O-]  C94307042 P147808042 | 1.292 |
| 19 | C[C@H]1[C@@H]([C@H]([C@H]([C@@H](O1)OC[C@@H]2[C@H]([C@@H]([C@H]([C@@H](O2)Oc3c(=O)c4c(cc(cc4oc3c5ccc(c(c5)OCCO)OCCO)OCCO)O)O)O)O)O)O)O  C85552699 P133897791 | 1.276 |
| 20 | CC(=O)Nc1ccc(cc1)S(=O)(=O)NNC(=O)CCC(=O)N[N-]S(=O)(=O)c2ccc(cc2)NC(=O)C  C98241107 P156253146 | 1.185 |
| 21 | Cc1cc(cc(c1O)[NH+](CC(=O)[O-])CC(=O)[O-])C2(c3ccccc3C(=O)O2)c4cc(c(c(c4)[NH+](CC(=O)[O-])CC(=O)[O-])O)C  C72099785…P111901397 | 1.185 |
| 22 | COC(=O)CNC(=O)[C@H](CSSC[C@@H](C(=O)NCC(=O)OC)NC(=O)CC[C@@H](C(=O)OC)N)NC(=O)CC[C@@H](C(=O)OC)[NH3+]  C77293156 P120588133 | 1.181 |
| 23 | c1nc(c2c(n1)n(cn2)[C@@H](CO)O[C@H](CO)CO[P@@](=O)([O-])O[P@@](=O)([O-])O[P@@](=O)(O)[O-])N  C44559534 P111216335 | 1.176 |
| 24 | c1cc(ccc1NC(=O)CN2CC[NH+](CC2)CCO)NC(=O)CN3CCN(CC3)CCO  C22941690 P83119194 | 1.145 |
| 25 | COC(=O)CNC(=O)[C@H](CSSC[C@@H](/C(=N/CC(=O)OC)/[O-])NC(=O)CC[C@@H](C(=O)OC)N)NC(=O)CC[C@@H](C(=O)OC)N  C77293156 P120588626 | 1.137 |
| 26 | CCOC(=O)C(CCC([NH3+])([P@@](=O)(O)[O-])[P@](=O)(O)[O-])(CCC([NH3+])([P@](=O)(O)[O-])[P@](=O)(O)[O-])C(=O)OCC  C97956277 P155544069 | 1.135 |
| 27 | CCOC(=O)C(=CNNC(=O)C(=O)NNC=C(C(=O)OCC)C(=O)OCC)C(=O)OCC  C15776619 P23781921 | 1.109 |
| 28 | COc1cc(ccc1O[C@H](CO)[C@H](c2ccc(c(c2)OC)O)O)CCCO[C@@H]3[C@@H]([C@@H]([C@@H]([C@@H](O3)CO)O)O)O  C72319945 P112401568 | 1.092 |
| 29 | c1cc(ccc1NC(=O)CC(=O)Nc2ccc(cc2)S(=O)(=O)NCCO)S(=O)(=O)NCCO  C04748117 P09431572 | 1.079 |
| 30 | C=C(CCO)C(=O)OC[C@H]1[C@@H]([C@@H]([C@H](CO1)OC(=O)C(=C)CCO[C@H]2[C@@H]([C@H]([C@@H]([C@H](O2)COC(=O)C(=C)CCO)O)O)O)O)O  C67911826 P104263419 | 1.067 |
| 31 | C=C(CCO)C(=O)OC[C@H]1[C@@H]([C@@H]([C@@H](CO1)OC(=O)C(=C)CCO[C@H]2[C@@H]([C@H]([C@@H]([C@H](O2)COC(=O)C(=C)CCO)O)O)O)O)O  C67911823 P104263415 | 1.067 |
| 32 | C=C(CCO)C(=O)OC[C@@H]1[C@@H]([C@@H]([C@H](CO1)OC(=O)C(=C)CCO[C@H]2[C@@H]([C@H]([C@@H]([C@H](O2)COC(=O)C(=C)CCO)O)O)O)O)O  C67911821 P104263412 | 1.067 |
| 33 | C=C(CCO)C(=O)OC[C@@H]1[C@@H]([C@@H]([C@@H](CO1)OC(=O)C(=C)CCO[C@H]2[C@@H]([C@H]([C@@H]([C@H](O2)COC(=O)C(=C)CCO)O)O)O)O)O  C67911819 P104263410 | 1.067 |
| 34 | c1cc(ccc1OC[C@H](C[NH2+]CCN2CCOCC2)O)OC[C@H](CNCCN3CCOCC3)O  C27529524 P40565095 | 1.066 |
| 35 | c1ccc(c(c1)C(=O)N[C@@H](CO)C(=O)N[C@@H](CC(=O)[O-])C(=O)N[C@@H](CCCCNc2ccc(cc2[N+](=O)[O-])[N+](=O)[O-])C(=O)N3CCC[C@H]3C(=O)[O-])N  C98224537 P156217278 | 1.043 |
| 36 | c1nc(c2c(n1)n(cn2)[C@@H](C=O)O[C@@H](CO[P@@](=O)([O-])O[P@@](=O)([O-])O[P@@](=O)(O)[O-])C=O)N  C90668375 P141806950 | 1.034 |
| 37 | CC(CC(=O)OCc1ccc(cc1)O[C@H]2[C@@H]([C@H]([C@@H]([C@H](O2)CO)O)O)O)(CC(=O)OCc3ccc(cc3)O[C@H]4[C@@H]([C@H]([C@@H]([C@H](O4)CO)O)O)O)O  C67911963 P104263624 | 1.017 |
| 38 | C=C(CCO)C(=O)OC[C@H]1[C@H]([C@@H]([C@@H](CO1)OC(=O)C(=C)CCO[C@H]2[C@@H]([C@H]([C@@H]([C@H](O2)CO)O)O)O)O)O  C77257478 P120526892 | 1.009 |
| 39 | CC(=O)O[C@H]1[C@H]([C@@H](O[C@@H]([C@@H]1O)O[C@@H](/C=N\Cc2ccc(cc2)O)[C@@H](CO)O[C@@H](/C=N/Cc3ccc(cc3)O)O)CO)O  C70670108 P109028532 | 0.966 |
| 40 | CC(=O)O[C@@H]1[C@H]([C@@H](O[C@@H]([C@@H]1O)O[C@@H](/C=N\Cc2ccc(cc2)O)[C@@H](CO)O[C@@H](/C=N/Cc3ccc(cc3)O)O)CO)O  C70670107 P109028531 | 0.966 |
| 41 | CC(=O)O[C@H]1[C@@H]([C@@H](O[C@@H]([C@@H]1O)O[C@@H](/C=N\Cc2ccc(cc2)O)[C@@H](CO)O[C@@H](/C=N/Cc3ccc(cc3)O)O)CO)O  C70670106 P109028530 | 0.966 |
| 42 | CC(=O)O[C@@H]1[C@@H]([C@@H](O[C@@H]([C@@H]1O)O[C@@H](/C=N\Cc2ccc(cc2)O)[C@@H](CO)O[C@@H](/C=N/Cc3ccc(cc3)O)O)CO)O  C70670105 P109028529 | 0.966 |
| 43 | CC(=O)OC[C@H]([C@H]([C@H]1[C@@H]([C@H](C[C@](O1)(C(=O)OC)O)OC(=O)C)[NH3+])OC(=O)C)OC(=O)C  C88465885 P138372311 | 0.963 |
| 44 | CC(=O)O[C@H]1[C@@H]([C@@H](O[C@@H]([C@@H]1O)O[C@@H](/C=N/c2ccc3c(c2)C(=O)NC3=O)[C@@H](CO)O[C@@H](/C=N/c4ccc5c(c4)C(=O)NC5=O)O)CO)O  C79204416 P123667679 | 0.949 |
| 45 | CC(=O)O[C@H]1[C@H]([C@@H](O[C@@H]([C@@H]1O)O[C@@H](C/N=C/c2ccccc2O)[C@@H](CO)O[C@@H](C/N=C/c3ccccc3O)O)CO)O  C70672775 P109035700 | 0.932 |
| 46 | CC(=O)O[C@@H]1[C@H]([C@@H](O[C@@H]([C@@H]1O)O[C@@H](C/N=C/c2ccccc2O)[C@@H](CO)O[C@@H](C/N=C/c3ccccc3O)O)CO)O  C70672772 P109035695 | 0.932 |
| 47 | CC(=O)O[C@H]1[C@@H]([C@@H](O[C@@H]([C@@H]1O)O[C@@H](C/N=C/c2ccccc2O)[C@@H](CO)O[C@@H](C/N=C/c3ccccc3O)O)CO)O  C70672769 P109035691 | 0.932 |
| 48 | CC(=O)O[C@@H]1[C@@H]([C@@H](O[C@@H]([C@@H]1O)O[C@@H](C/N=C/c2ccccc2O)[C@@H](CO)O[C@@H](C/N=C/c3ccccc3O)O)CO)O  C70672768 P109035686 | 0.932 |
| 49 | C(C[NH+](CC(=O)NCC(=O)N)CC(=O)[O-])[NH+](CC[NH+](CC(=O)NCC(=O)N)CC(=O)[O-])CC(=O)[O-]  C22942290 P66135663 | 0.928 |
| 50 | c1ccc(cc1)CO[C@H]2[C@@H]([C@H]([C@@H]([C@H](O2)CO)O)O[C@H]3[C@@H]([C@H]([C@@H]([C@H](O3)CO)O)O)O)O[C@H]4[C@@H]([C@](CO4)  (CO)O)O  C77269244 P120545333 | 0.907 |
| 51 | CN(Cc1cnc2c(n1)c(nc([nH+]2)N)N)c3ccc(cc3)C(=O)N[C@H](CCC(=O)NCCNC(=O)CCCC[C@H]4[C@@H]5[C@H](CS4)NC(=O)N5)C(=O)[O-]  C72131520 P111978842 | 0.905 |
| 52 | CN(Cc1cnc2c(n1)c(nc([nH+]2)N)N)c3ccc(cc3)C(=O)N[C@@H](CCC(=O)NCCNC(=O)CCCC[C@H]4[C@@H]5[C@H](CS4)NC(=O)N5)C(=O)[O-]  C72131519 P111978840 | 0.905 |
| 53 | COCC(=O)N[C@H]1CCN([C@@H](C1)C(=O)N[C@H](Cc2ccccc2)C(=O)N)C(=O)COC  C03838450 P04499823 | 0.895 |
| 54 | c1ccc(cc1)COC(=O)NCC(=O)N2CCC[C@H]2C(=O)N[C@@H](CCCNC(=[NH2+])N)C(=O)Nc3ccc(cc3)[N+](=O)[O-]  C71788471 P111270671 | 0.881 |
| 55 | c1cc(c(cc1F)OCCOc2cc(ccc2N(CC(=O)N)CC(=O)N)F)N(CC(=O)N)CC(=O)N  C65356700 P100463509 | 0.879 |
| 56 | C[S@](=O)CCCC/C(=N/OS(=O)(=O)[O-])/S[C@H]1[C@@H]([C@H]([C@@H]([C@H](O1)CO)O)O)O  C30160165 P44297720 | 0.877 |
| 57 | C1CS(=O)(=O)C[C@H]1[NH2+]COC[C@H](COCN[C@H]2CCS(=O)(=O)C2)OCN[C@@H]3CCS(=O)(=O)C3  C40168182 P60966418 | 0.858 |
| 58 | C1CS(=O)(=O)C[C@@H]1[NH2+]COC[C@H](COCN[C@H]2CCS(=O)(=O)C2)OCN[C@@H]3CCS(=O)(=O)C3  C40168180 P60966414 | 0.858 |
| 59 | CC(=O)O[C@H]1[C@H]([C@@H](O[C@@H]([C@@H]1O)O[C@@H](C/N=C/c2ccccc2O)[C@@H](CO)O[C@@H](C/[NH+]=C/c3ccccc3O)O)CO)O  C70672775 P109035300 | 0.854 |
| 60 | CC(=O)O[C@@H]1[C@H]([C@@H](O[C@@H]([C@@H]1O)O[C@@H](C/N=C/c2ccccc2O)[C@@H](CO)O[C@@H](C/[NH+]=C/c3ccccc3O)O)CO)O  C70672772 P109035293 | 0.854 |
| 61 | CC(=O)O[C@H]1[C@@H]([C@@H](O[C@@H]([C@@H]1O)O[C@@H](C/N=C/c2ccccc2O)[C@@H](CO)O[C@@H](C/[NH+]=C/c3ccccc3O)O)CO)O  C70672769 P109035285 | 0.854 |
| 62 | CC(=O)O[C@@H]1[C@@H]([C@@H](O[C@@H]([C@@H]1O)O[C@@H](C/N=C/c2ccccc2O)[C@@H](CO)O[C@@H](C/[NH+]=C/c3ccccc3O)O)CO)O  C70672768 P109035277 | 0.854 |
| 63 | c1cc(ccc1CCOC(=O)C2=CO[C@H](/C(=C\CO)/[C@@H]2CC(=O)OCCc3ccc(c(c3)O)O)O[C@H]4[C@@H]([C@H]([C@@H]([C@H](O4)CO)O)O)O)O  C67911283 P104262618 | 0.848 |
| 64 | CC(C)C[C@@]([C@@H](C(=O)OCc1ccc(cc1)O[C@H]2[C@@H]([C@H]([C@@H]([C@H](O2)CO)O)O)O)O)(C(=O)OCc3ccc(cc3)O[C@H]4[C@@H]([C@H]([C@@H]([C@H](O4)CO)O)O)O)O  C95788020 P150692613 | 0.847 |
| 65 | CC(C)[C@@](CCO[C@H]1[C@@H]([C@H]([C@@H]([C@H](O1)CO[C@@H]2[C@@H]([C@](CO2)(CO)O)O)O)O)O)(CO)O  C77257444 P120526827 | 0.843 |
| 66 | c1ccc(cc1)COC(=O)NCC(=O)NCC(=O)Nc2ccn(c(=O)n2)[C@@H]3[C@@H]([C@@H]([C@@H](O3)CO)O)O  C35088781 P52081444 | 0.830 |
| 67 | CC(=O)O[C@H]1[C@H](O[C@@H]([C@@H]([C@H]1OC(=O)C)OC(=O)C)O[C@]2([C@H]([C@@H]([C@H](O2)CO)OC(=O)C)OC(=O)C)CO)CO  C77312245 P120616876 | 0.824 |
| 68 | c1cc(c(cc1CCOC(=O)C[C@@H]\2C(=CO[C@H](/C2=C\CO)O[C@H]3[C@@H]([C@H]([C@@H]([C@H](O3)CO)O)O)O)C(=O)OCCc4ccc(c(c4)O)O)O)O  C67911279 P104262611 | 0.813 |
| 69 | CC(=O)O[C@H]1[C@H]([C@@H](O[C@@H]([C@@H]1O)O[C@@H](/C=N\Cc2ccccc2O)[C@@H](CO)O[C@@H](/C=N/Cc3ccccc3O)O)CO)O  C70672682 P109033136 | 0.782 |
| 70 | CC(=O)O[C@@H]1[C@H]([C@@H](O[C@@H]([C@@H]1O)O[C@@H](/C=N\Cc2ccccc2O)[C@@H](CO)O[C@@H](/C=N/Cc3ccccc3O)O)CO)O  C70672677 P109033129 | 0.782 |
| 71 | CC(=O)O[C@H]1[C@@H]([C@@H](O[C@@H]([C@@H]1O)O[C@@H](/C=N\Cc2ccccc2O)[C@@H](CO)O[C@@H](/C=N/Cc3ccccc3O)O)CO)O  C70672674 P109033125 | 0.782 |
| 72 | CC(=O)O[C@@H]1[C@@H]([C@@H](O[C@@H]([C@@H]1O)O[C@@H](/C=N\Cc2ccccc2O)[C@@H](CO)O[C@@H](/C=N/Cc3ccccc3O)O)CO)O  C70672673 P109033124 | 0.782 |
| 73 | C=C[C@@H]1[C@@H](C(=CO[C@@H]1O[C@@H]2[C@@H]([C@@H]([C@@H]([C@@H](O2)CO)O)O)O)C(=O)[O])CCOC(=O)c3cc(c(c(c3)[O])O)O  C67912601 P104267119 | 0.769 |
| 74 | c1ccc(c(c1)C(=O)N/N=C/[C@@H]([C@@H]([C@@H](COC[C@@H]2[C@@H]([C@@H]([C@@H]([C@@H](O2)CO)O)O)O)[C@@H](CO)O)O)O)[O-]  C33897335 P54072373 | 0.759 |
| 75 | COCc1nnc(o1)CSc2nnc(n2N)N/N=C/c3cc(c(c(c3)OC)OC)OC  C20920227 P31234736 | 0.736 |
| 76 | CC(=O)O[C@H]1[C@H]([C@@H](O[C@@H]([C@@H]1O)O[C@@H](C/[NH+]=C/c2ccccc2O)[C@@H](CO)O[C@@H](C/N=C/c3ccccc3O)O)CO)O  C70672775 P109035297 | 0.733 |
| 77 | CC(=O)O[C@@H]1[C@H]([C@@H](O[C@@H]([C@@H]1O)O[C@@H](C/[NH+]=C/c2ccccc2O)[C@@H](CO)O[C@@H](C/N=C/c3ccccc3O)O)CO)O  C70672772 P109035289 | 0.733 |
| 78 | CC(=O)O[C@H]1[C@@H]([C@@H](O[C@@H]([C@@H]1O)O[C@@H](C/[NH+]=C/c2ccccc2O)[C@@H](CO)O[C@@H](C/N=C/c3ccccc3O)O)CO)O  C70672769 P109035281 | 0.733 |
| 79 | CC(=O)O[C@@H]1[C@@H]([C@@H](O[C@@H]([C@@H]1O)O[C@@H](C/[NH+]=C/c2ccccc2O)[C@@H](CO)O[C@@H](C/N=C/c3ccccc3O)O)CO)O  C70672768 P109035273 | 0.733 |
| 80 | CCCCCCCCO[C@H]1[C@H]([C@H]([C@@H]([C@H](O1)CO[C@@H]2[C@H]([C@H]([C@@H]([C@H](O2)CO)O)O)O)O)O[C@@H]3[C@H]([C@H]([C@@H]([C@H](O3)CO)O)O)O)O  C77311964 P120616466 | 0.726 |
| 81 | CCCCCCCCO[C@H]1[C@@H]([C@H]([C@@H]([C@H](O1)CO)O)O)O[C@@H]2[C@H]([C@H]([C@@H]([C@H](O2)CO)O)O)O[C@H]3[C@@H]([C@H]([C@@H]([C@H](O3)CO)O)O)NC(=O)C  C77311957 P120616455 | 0.726 |
| 82 | CC(=O)OC[C@@H]1[C@H]([C@@H]([C@H]([C@H](O1)O[C@]2([C@H]([C@@H]([C@H](O2)CO)OC(=O)/C=C/c3ccccc3)O)CO)O)O)O  C67911755 P104263322 | 0.697 |
| 83 | COC(=O)CNC(=O)[C@H](CSSC[C@@H](C(=O)NCC(=O)OC)NC(=O)CC[C@@H](C(=O)OC)N)NC(=O)CC[C@@H](C(=O)OC)N  C77293156 P120586988 | 0.668 |
| 84 | CCOC(=O)C1=C(NC(=C([C@H]1c2ccccc2Cl)C(=O)OC)C)COCC[NH2+][C@H]3[C@@H]([C@H]([C@@H]([C@H](O3)CO)O[C@H]4[C@@H]([C@H]([C@H]([C@H](O4)CO)O)O)O)O)O  C77271546 P120551391 | 0.655 |
| 85 | CCOC(=O)C1=C(NC(=C([C@@H]1c2ccccc2Cl)C(=O)OC)C)COCC[NH2+][C@H]3[C@@H]([C@H]([C@@H]([C@H](O3)CO)O[C@H]4[C@@H]([C@H]([C@H]([C@H](O4)CO)O)O)O)O)O  C77271542 P120551385 | 0.655 |
| 86 | CCOC(=O)C1=C(NC(=C([C@H]1c2ccccc2Cl)C(=O)OC)C)COCC[NH2+][C@@H]3[C@@H]([C@H]([C@@H]([C@H](O3)CO)O[C@H]4[C@@H]([C@H]([C@H]([C@H](O4)CO)O)O)O)O)O  C77271535 P120551377 | 0.655 |
| 87 | CCOC(=O)C1=C(NC(=C([C@@H]1c2ccccc2Cl)C(=O)OC)C)COCC[NH2+][C@@H]3[C@@H]([C@H]([C@@H]([C@H](O3)CO)O[C@H]4[C@@H]([C@H]([C@H]([C@H](O4)CO)O)O)O)O)O  C77271526 P120551373 | 0.655 |
| 88 | C(CC(=O)N(CC(=O)NCCO)CC(=O)NCCO)C(=O)N(CC(=O)NCCO)CC(=O)NCCO  C13756556 P21182593 | 0.625 |
| 89 | CC(C)C[C@@]([C@@H](C(=O)OCc1ccc(cc1)O[C@H]2[C@@H]([C@H]([C@@H]([C@H](O2)CO)O)O)O)[O])(C(=O)OCc3ccc(cc3)O[C@H]4[C@@H]([C@H]([C@@H]([C@H](O4)CO)O)O)O)O  C95788020 P150692684 | 0.587 |
| 90 | CC(=O)O[C@H]1[C@H]([C@@H](O[C@@H]([C@@H]1O)O[C@@H](C/[NH+]=C/c2ccccc2O)[C@@H](CO)O[C@@H](C/[NH+]=C/c3ccccc3O)O)CO)O  C70672775 P109033271 | 0.578 |
| 91 | CC(=O)O[C@H]1[C@H]([C@@H](O[C@@H]([C@@H]1O)O[C@@H](C/[NH+]=C/c2ccccc2O)[C@@H](CO)O[C@@H](C/[NH+]=C/c3ccccc3O)O)CO)O  C70672775..P109033271 | 0.578 |
| 92 | CC(=O)O[C@@H]1[C@H]([C@@H](O[C@@H]([C@@H]1O)O[C@@H](C/[NH+]=C/c2ccccc2O)[C@@H](CO)O[C@@H](C/[NH+]=C/c3ccccc3O)O)CO)O  C70672772 P109033267 | 0.578 |
| 93 | CC(=O)O[C@H]1[C@@H]([C@@H](O[C@@H]([C@@H]1O)O[C@@H](C/[NH+]=C/c2ccccc2O)[C@@H](CO)O[C@@H](C/[NH+]=C/c3ccccc3O)O)CO)O  C70672769 P109033263 | 0.578 |
| 94 | CC(=O)O[C@@H]1[C@@H]([C@@H](O[C@@H]([C@@H]1O)O[C@@H](C/[NH+]=C/c2ccccc2O)[C@@H](CO)O[C@@H](C/[NH+]=C/c3ccccc3O)O)CO)O  C70672768 P109033261 | 0.578 |
| 95 | CC(=O)O[C@H]1[C@@H]([C@H](O[C@H]([C@@H]1OC(=O)C)[n+]2c(nc(c(n2)c3cccc(c3Cl)Cl)N)N)C(=O)OC)OC(=O)C  C77301742 P120598486 | 0.570 |
| 96 | C(C[NH+](CC(=O)NCC(=O)N)CC(=O)[O-])[NH+](CCN(CC(=O)NCC(=O)N)CC(=O)[O-])CC(=O)[O-]  C22942290 P66135664 | 0.494 |
| 97 | C/C(=C\C(=O)NCCC[C@H]1C(=O)N[C@H](C(=O)N1)CCCNC(=O)/C=C(\C)/CCO[C@@H]2[C@@H]([C@@H]([C@@H]([C@@H](O2)CO)O)O)O)/CCO  C72320094 P112401766 | 0.451 |
| 98 | C/C(=C\C(=O)NCCC[C@@H]1C(=O)N[C@H](C(=O)N1)CCCNC(=O)/C=C(\C)/CCO[C@@H]2[C@@H]([C@@H]([C@@H]([C@@H](O2)CO)O)O)O)/CCO  C72320093 P112401765 | 0.451 |
| 99 | C/C(=C\C(=O)NCCC[C@H]1C(=O)N[C@@H](C(=O)N1)CCCNC(=O)/C=C(\C)/CCO[C@@H]2[C@@H]([C@@H]([C@@H]([C@@H](O2)CO)O)O)O)/CCO  C72320092 P112401764 | 0.451 |
| 100 | C/C(=C\C(=O)NCCC[C@@H]1C(=O)N[C@@H](C(=O)N1)CCCNC(=O)/C=C(\C)/CCO[C@@H]2[C@@H]([C@@H]([C@@H]([C@@H](O2)CO)O)O)O)/CCO  C72320091 P112401763 | 0.451 |
| 101 | C(CN(CC[NH+](CC(=O)NCC(=O)N)CC(=O)[O-])CC(=O)[O-])[NH+](CC(=O)NCC(=O)N)CC(=O)[O-]  C22942290 P66135590 | 0.439 |
| 102 | CC(C)(C)OC(=O)N[C@H](CC(=O)OCc1ccccc1)C(=O)N2CCC[C@@H]2C(=O)N[C@@H](CCCNC(=[NH2+])N)C(=O)[O-]  C70665815 P109013667 | 0.426 |
| 103 | CC(C)(C)OC(=O)N[C@@H](CC(=O)OCc1ccccc1)C(=O)N2CCC[C@@H]2C(=O)N[C@@H](CCCNC(=[NH2+])N)C(=O)[O-]  C70665813 P109013664 | 0.426 |
| 104 | c1nc(c2c(n1)n(cn2)[C@H]3[C@@H]([C@@H]([C@H](O3)CO[P@@](=O)(O)[O-])O)OP(=O)([O-])[O-])NCCCCCC[NH3+]  C60291548 P92076726 | 0.425 |
| 105 | C[C@@H]1[C@H]([C@@H]([C@@H]([C@H](O1)OC[C@H]2[C@@H]([C@H]([C@@H]([C@H](O2)Oc3c(=O)c4c(cc(cc4oc3c5ccc(c(c5)OCCO)OCCO)OCCO)[O-])O)O)O)O)O)O C  94313260 P147822206 | 0.415 |
| 106 | C[C@H]1[C@@H]([C@H]([C@H]([C@@H](O1)OC[C@@H]2[C@H]([C@@H]([C@H]([C@@H](O2)Oc3c(=O)c4c(cc(cc4oc3c5ccc(c(c5)OCCO)OCCO)OCCO)[O-])O)O)O)O)O)O C  85552699 P133917388 | 0.415 |
| 107 | COc1cc(ccc1O)C(=O)OC[C@]2(CO[C@@H]([C@@H]2O)OC[C@@H]3[C@H]([C@@H]([C@H]([C@@H](O3)Oc4ccc(c(c4)OC)O)O)O)O)O  C38139658 P57374077 | 0.414 |
| 108 | C1[C@H]2[C@@H]([C@@H](S1)CCCCC(=O)NCCSSCCC(=O)N[C@@H](C(=O)NCCCC(=O)O)C(=O)NCCCC(=O)[O-])NC(=O)N2  C72130439 P111976122 | 0.369 |
| 109 | Cc1nnc(o1)CSc2nnc(s2)NC(=O)CSc3nc([nH]n3)N/N=C/c4ccncc4  C20837717 P31090964 | 0.330 |
| 110 | c1cc(ccc1CCOC(=O)C[C@@H]\2C(=CO[C@H](/C2=C\CO)O[C@H]3[C@@H]([C@H]([C@@H]([C@H](O3)CO)O)O)O)C(=O)OCCc4ccc(c(c4)O)O)O  C67911281 P104262615 | 0.326 |
| 111 | c1cc(ccc1C[C@H](C(=O)N[C@H](CSSC[C@@H](C(=O)N)NC(=O)[C@H](Cc2ccc(cc2)O)N)C(=O)N)N)O  C71788315 P111271000 | 0.281 |
| 112 | COCCNc1[n+](c(cs1)c2ccc(c(c2)C(=O)N)[O-])/N=C/c3cc(c(c(c3)OC)OC)OC  C97993839 P155638880 | 0.217 |
| 113 | CC(C)(CO[P@@](=O)([O])O[P@@](=O)([O])OC[C@@H]1[C@@H]([C@@H]([C@@H](O1)n2cnc3c2ncnc3N)O)O)[C@@H](C(=O)NCCC(=O)NCCS)O  C62227870 P95488681 | 0.199 |
| 114 | C1[C@H]2[C@@H]([C@@H](S1)CCCCC(=O)NCCCCCC(=O)NCCCCCNC(=O)[C@H](CCCNC(=S)N)N)NC(=O)N2  C72130428 P111976071 | 0.111 |
| 115 | CCCCOC(=O)[C@@](CC[NH+]=C(N)N)(C(=O)[C@@H](Cc1ccccc1)N)[C@@](C(=O)[C@@H]2CCC[NH2+]2)(C(=O)[O-])N  C98045450 P155774399 | 0.094 |
| 116 | COc1cc(ccc1O)/C=C/C(=O)OC[C@@H]2[C@H]([C@@H]([C@](O2)(CO)O[C@@H]3[C@@H]([C@H]([C@@H]([C@H](O3)CO)O)O)O)OC(=O)/C=C/c4ccc(c(c4)OC)O)O  C77257397 P120526726 | 0.052 |
| 117 | CC(=O)O[C@@H]1[C@@H]([C@@H](O[C@@H]([C@@H]1O)O[C@@H](/C=N\C23CN4CN(C2)CN(C3)C4)[C@@H](CO)O[C@@H](/C=N/C56CN7CN(C5)CN(C6)C7)O)CO)O  C79209987 P123677403 | 0.036 |
| 118 | C[C@@](CO[C@H]1[C@@H]([C@H]([C@@H]([C@H](O1)COC(=O)/C=C/c2cc(c(c(c2)OC)O)OC)O)O)O)([C@H](CO)O)O  C72320370 P112402092 | -0.083 |
| 119 | CCC(=O)OCCCCCCCCO[C@H]1[C@@H]([C@H]([C@@H]([C@H](O1)CO)O[C@H]2[C@@H]([C@H]([C@@H]([C@H](O2)CO)O)O)NC(=O)C)O)NC(=O)C  C77292230 P120585584 | N.M.*^a^* |
| 120 | CC(C)C[C@@H](C(=O)N1CCC[C@H]1C(=O)N[C@@H](Cc2ccccc2)C(=O)N[C@@H](Cc3ccccc3)C(=O)N[C@@H](CC(=O)O)C(=O)[O-])[NH3+]  C98229901 P156228100 | N.M. |
| 121 | CCCCOC(=O)[C@](CC[NH+]=C(N)N)(C(=O)[C@@H](Cc1ccccc1)N)[C@@](C(=O)[C@@H]2CCC[NH2+]2)(C(=O)[O-])N  C98045451 P155774400 | N.M. |
| 122 | C1[C@H]2[C@@H]([C@@H](S1)CCCCC(=O)NCCCCCSC[C@@H](C(=O)NCC(=O)[O-])NC(=O)CC[C@@H](C(=O)[O-])N)NC(=O)N2  C77312279 P120619636 | N.M. |
| 123 | C1[C@@H]2[C@@H]([C@@H](S1)CCCCC(=O)NCCCCCSC[C@@H](C(=O)NCC(=O)[O-])NC(=O)CC[C@@H](C(=O)[O-])N)NC(=O)N2  C77312275 P120619634 | N.M. |
| 124 | CCCCOC(=O)[C@@](CC[NH+]=C(N)N)(C(=O)[C@@H](Cc1ccccc1)N)[C@@](C(=O)[C@@H]2CCCN2)(C(=O)[O-])N  C98045450 P155774490 | N.M. |
| 125 | CCCCOC(=O)[C@](CC[NH+]=C(N)N)(C(=O)[C@@H](Cc1ccccc1)N)[C@@](C(=O)[C@@H]2CCCN2)(C(=O)[O-])N  C98045451 P155774491 | N.M. |
| 126 | CCCCOC(=O)[C@@](CC[NH+]=C(N)N)(C(=O)[C@H](Cc1ccccc1)N)[C@@](C(=O)[C@@H]2CCCN2)(C(=O)[O-])N  C98045452 P155774492 | N.M. |
| 127 | CCCCOC(=O)[C@](CC[NH+]=C(N)N)(C(=O)[C@H](Cc1ccccc1)N)[C@@](C(=O)[C@@H]2CCCN2)(C(=O)[O-])N  C98045453 P155774493 | N.M. |
| 128 | c1ccc(cc1)C[C@H](C(=O)N[C@H](CC(=O)[O-])C(=O)N[C@@H](CO)C(=O)[O-])NC(=O)[C@@H](CCC[NH+]=C(N)N)N  C33793872 P155768550 | N.M. |
| 129 | c1ccc(cc1)C[C@@H](C(=O)N[C@H](CC(=O)[O-])C(=O)N[C@@H](CO)C(=O)[O-])NC(=O)[C@@H](CCC[NH+]=C(N)N)N  C33793871 P155768549 | N.M. |
| 130 | c1ccc(cc1)C[C@H](C(=O)N[C@@H](CC(=O)[O-])C(=O)N[C@@H](CO)C(=O)[O-])NC(=O)[C@@H](CCC[NH+]=C(N)N)N  C33793870 P155768548 | N.M. |
| 131 | c1ccc(cc1)C[C@@H](C(=O)N[C@@H](CC(=O)[O-])C(=O)N[C@@H](CO)C(=O)[O-])NC(=O)[C@@H](CCC[NH+]=C(N)N)N  C33793869 P155768547 | N.M. |
| 132 | CCCCOC(=O)[C@@](CC[NH+]=C(N)N)(C(=O)[C@H](Cc1ccccc1)N)[C@@](C(=O)[C@@H]2CCC[NH2+]2)(C(=O)[O-])N  C98045452 P155774401 | N.M. |
| 133 | CCCCOC(=O)[C@](CC[NH+]=C(N)N)(C(=O)[C@H](Cc1ccccc1)N)[C@@](C(=O)[C@@H]2CCC[NH2+]2)(C(=O)[O-])N  C98045453 P155774402 | N.M. |
| 134 | C[C@H]1[C@@H]([C@H]([C@H]([C@@H](O1)O[C@H]2[C@@H]3C=CO[C@H]([C@@H]3[C@@]4([C@H]2O4)CO)O[C@H]5[C@@H]([C@H]([C@@H]([C@H](O5)CO)O)O)O)OC(=O)/C=C/c6ccc(cc6)O)O)O  C95785971 P150682708 | N.M. |
| 135 | c1c(c(c(c[n+]1CCCC[C@H](C(=O)[O-])[NH3+])CC[C@H](C(=O)[O-])N)CCC[C@@H](C(=O)[O-])N)CC[C@@H](C(=O)[O-])[NH3+]  C35024535 P111217451 | N.M. |
| 136 | c1c(c(c(c[n+]1CCCC[C@@H](C(=O)[O-])[NH3+])CC[C@H](C(=O)[O-])N)CCC[C@@H](C(=O)[O-])N)CC[C@@H](C(=O)[O-])[NH3+]  C35024532 P111217450 | N.M. |
| 137 | c1c(c(c(c[n+]1CCCC[C@H](C(=O)[O-])[NH3+])CC[C@@H](C(=O)[O-])N)CCC[C@@H](C(=O)[O-])N)CC[C@@H](C(=O)[O-])[NH3+]  C35024530 P111217449 | N.M. |
| 138 | c1c(c(c(c[n+]1CCCC[C@H](C(=O)[O-])[NH3+])CC[C@@H](C(=O)[O-])[NH3+])CCC[C@@H](C(=O)[O-])[NH3+])CC[C@H](C(=O)[O-])[NH3+]  C35024535 P111217444 | N.M. |
| 139 | c1c(c(c(c[n+]1CCCC[C@@H](C(=O)[O-])[NH3+])CC[C@@H](C(=O)[O-])[NH3+])CCC[C@@H](C(=O)[O-])[NH3+])CC[C@H](C(=O)[O-])[NH3+]  C35024532 P111217443 | N.M. |
| 140 | c1c(c(c(c[n+]1CCCC[C@H](C(=O)[O-])[NH3+])CC[C@@H](C(=O)[O-])[NH3+])CCC[C@@H](C(=O)[O-])[NH3+])CC[C@@H](C(=O)[O-])[NH3+]  C35024530 P111217442 | N.M. |
| 141 | c1c(c(c(c[n+]1CCCC[C@H](C(=O)[O-])N)CC[C@H](C(=O)[O-])N)CCC[C@@H](C(=O)[O-])N)CC[C@@H](C(=O)[O-])[NH3+]  C35024535 P111217399 | N.M. |
| 142 | c1c(c(c(c[n+]1CCCC[C@@H](C(=O)[O-])N)CC[C@H](C(=O)[O-])N)CCC[C@@H](C(=O)[O-])N)CC[C@@H](C(=O)[O-])[NH3+]  C35024532 P111217398 | N.M. |
| 143 | c1c(c(c(c[n+]1CCCC[C@H](C(=O)[O-])N)CC[C@@H](C(=O)[O-])N)CCC[C@@H](C(=O)[O-])N)CC[C@@H](C(=O)[O-])[NH3+]  C35024530 P111217397 | N.M. |
| 144 | CCOC(=O)C(=O)/C(=N\Nc1c(nc[nH]1)C(=O)NC)/C(=O)N/N=C/c2ccc(c(c2)OC)O  C08385572 P12677418 | N.M. |
| 145 | CCOC(=O)C(=O)/C(=N\Nc1c([nH+]c[nH]1)C(=O)N)/C(=O)N/N=C/c2ccc(cc2)OC  C08385576 P12778271 | N.M. |
| 146 | c1ccc(c(c1)OCCOCCOc2ccccc2OCC(=O)NN)OCC(=O)NN  C15724455 P23715818 | N.M. |
| 147 | c1c(c(c(c[n+]1CCCC[C@H](C(=O)[O-])[NH3+])CC[C@@H](C(=O)[O-])N)CCC[C@@H](C(=O)[O-])[NH3+])CC[C@@H](C(=O)[O-])[NH3+]  C35024530 P51988123 | N.M. |
| 148 | c1c(c(c(c[n+]1CCCC[C@@H](C(=O)[O-])[NH3+])CC[C@@H](C(=O)[O-])N)CCC[C@@H](C(=O)[O-])[NH3+])CC[C@@H](C(=O)[O-])[NH3+]  C35024527 P51988120 | N.M. |
| 149 | C[C@H](C[C@H](N([C@@H](C[C@@H](C)O)O)C(=S)N([C@@H](C[C@@H](C)O)O)[C@@H](C[C@@H](C)O)O)O)O  C35188402 P52217208 | N.M. |
| 150 | CC1(C(=O)N(C(=O)N1CN2C(=O)N(C(=O)C2(C)C)C[C@H](CN(C)C)O)C[C@H](C[NH+](C)C)O)C  C09057268 P56461045 | N.M. |
| 151 | C1CS(=O)(=O)C[C@H]1CC(=O)NNC(=O)CSc2nnc(n2C3CC3)CCC(=O)N  C12777204 P60351380 | N.M. |
| 152 | C[C@H](C[C@H](N([C@@H](C[C@@H](C)O)O)C(=S)N([C@@H](C[C@@H](C)O)O)[C@@H](C[C@H](C)O)O)O)O  C35188400 P66135317 | N.M. |
| 153 | c1c(c(c(c[n+]1CCCC[C@H](C(=O)[O-])[NH3+])CC[C@H](C(=O)[O-])N)CCC[C@@H](C(=O)[O-])[NH3+])CC[C@@H](C(=O)[O-])[NH3+]  C35024535 P66592704 | N.M. |
| 154 | c1c(c(c(c[n+]1CCCC[C@@H](C(=O)[O-])[NH3+])CC[C@H](C(=O)[O-])N)CCC[C@@H](C(=O)[O-])[NH3+])CC[C@@H](C(=O)[O-])[NH3+]  C35024532 P66592700 | N.M. |
| 155 | c1c(c(c(c[n+]1CCCC[C@@H](C(=O)[O-])[NH3+])CC[C@@H](C(=O)[O-])[NH3+])CCC[C@@H](C(=O)[O-])[NH3+])CC[C@@H](C(=O)[O-])[NH3+]  C35024527 P90244258 | N.M. |
| 156 | c1c(c(c(c[n+]1CCCC[C@@H](C(=O)[O-])[NH3+])CC[C@@H](C(=O)[O-])[NH3+])CCC[C@@H](C(=O)[O-])[NH3+])CC[C@@H](C(=O)[O-])[NH3+]  C35024527 P90244258 | N.M. |
| 157 | c1c(c(c(c[n+]1CCCC[C@@H](C(=O)[O-])[NH3+])CC[C@@H](C(=O)[O-])N)CCC[C@@H](C(=O)[O-])N)CC[C@@H](C(=O)[O-])[NH3+]  C35024527 P90244903 | N.M. |
| 158 | CC(C)[C@@H](C(=O)OCCOCn1cnc2c1nc([nH]c2=O)NCNc3[nH]c(=O)c4c(n3)n(cn4)COCCOC(=O)[C@H](C(C)C)[NH3+])[NH3+]  C72130492 P111975966 | N.M. |
| 159 | CC(C)[C@@H](C(=O)OCCOCn1cnc2c1nc([nH]c2=O)NCNc3[nH]c(=O)c4c(n3)n(cn4)COCCOC(=O)[C@H](C(C)C)N)N  C72130492 P111975967 | N.M. |
| 160 | C1[C@H]2[C@H]([C@@H](S1)CCCCC(=O)NCCCCCSC[C@@H](C(=O)NCC(=O)[O-])NC(=O)CC[C@@H](C(=O)[O-])N)NC(=O)N2  C77312285 P120619645 | N.M. |
| 161 | C1[C@@H]2[C@H]([C@@H](S1)CCCCC(=O)NCCCCCSC[C@@H](C(=O)NCC(=O)[O-])NC(=O)CC[C@@H](C(=O)[O-])N)NC(=O)N2  C77312281 P120619640 | N.M. |
| 162 | c1ccc(cc1)CS(=O)(=O)N[C@H](CCCNC(=[NH2+])N)C(=O)N(CC(=O)N)[C@@H](CCCNC(=[NH2+])N)C=O  C83317970 P130273175 | N.M. |
| 163 | COCCNc1[n+](c(cs1)c2ccc(c(c2)C(=O)N)O)/N=C\c3cc(c(c(c3)OC)OC)OC  C97993838 P155638156 | N.M. |
| 164 | CCCCCCO[C@H]1[C@H]([C@@H]([C@@H]([C@@H](O1)CO)O[C@@H]2[C@@H]([C@@H]([C@@H]([C@@H](O2)CO)O)O)O)O)O  C98088217 P155907742 | N.M. |
| 165 | C1CCC(CC1)CCCCO[C@@H]2[C@H]([C@@H]([C@@H]([C@@H](O2)CO)O[C@@H]3[C@@H]([C@@H]([C@@H]([C@@H](O3)CO)O)O)O)O)O  C43193643 P155915139 | N.M. |
| 166 | c1nc(c2c(n1)n(cn2)[C@H]3[C@@H]([C@@H]([C@H](O3)COP(=O)([O-])[O-])OP(=O)([O-])[O-])O)NCCCCCC[NH3+]  C98216486 P156200137 | N.M. |
| 167 | COC(=O)[C@H](Cc1ccc(cc1)OC(=O)CCC(=O)O[C@@H]2[C@@H]3[C@@H](CO[P@@](=O)(O3)[O-])O[C@@H]2n4cnc5c4ncnc5N)N  C04517756 P156200154 | N.M. |
| 168 | c1ccc(cc1)C[C@H](C(=O)N[C@@H](CO)C(=O)N2CCC[C@@H]2C(=O)[O])NC(=O)CNC(=O)[C@@H]3CCCN3C(=O)[C@@H]4CCCN4C(=O)[C@@H](CCCNC(=[NH2+])N)[NH3+]  C98217025 P156201654 | N.M. |
| 169 | c1ccc(cc1)C[C@@H](C(=O)N[C@@H](CO)C(=O)N2CCC[C@@H]2C(=O)[O])NC(=O)CNC(=O)[C@@H]3CCCN3C(=O)[C@@H]4CCCN4C(=O)[C@@H](CCCNC(=[NH2+])N)[NH3+]  C98217022 P156201651 | N.M. |
| 170 | c1ccc2cc(ccc2c1)NC(=O)[C@H](CSSC[C@@H](C(=O)Nc3ccc4ccccc4c3)NC(=O)CC[C@@H](C(=O)[O-])N)NC(=O)CC[C@@H](C(=O)[O-])N  C98216813 P156201843 | N.M. |
| 171 | c1ccc2cc(ccc2c1)NC(=O)[C@H](CSSC[C@@H](C(=O)Nc3ccc4ccccc4c3)NC(=O)CC[C@@H](C(=O)[O-])N)NC(=O)CC[C@@H](C(=O)[O-])[NH3+]  C98216813 P156201841 | N.M. |
| 172 | c1ccc(cc1)C[C@H](C(=O)N[C@@H](CO)C(=O)N2CCC[C@@H]2C(=O)[O])NC(=O)CNC(=O)[C@@H]3CCCN3C(=O)[C@@H]4CCCN4C(=O)[C@@H](CCCNC(=[NH2+])N)N  C98217025 P156201911 | N.M. |
| 173 | c1ccc(cc1)C[C@@H](C(=O)N[C@@H](CO)C(=O)N2CCC[C@@H]2C(=O)[O])NC(=O)CNC(=O)[C@@H]3CCCN3C(=O)[C@@H]4CCCN4C(=O)[C@@H](CCCNC(=[NH2+])N)N  C98217022 P156201908 | N.M. |

*^a^*N.M.: no match: for the compounds labelled by N.M. no matching with at least three features of SUB1-PHA was found.
